# Integrative multi-omics investigation of sleep apnea: gut microbiome metabolomics, proteomics and phenome-wide association study

**DOI:** 10.1186/s12986-025-00925-0

**Published:** 2025-06-10

**Authors:** Shuxu Wei, Ronghuai Shen, Xiaojia Lu, Xinyi Li, Lingbin He, Youti Zhang, Xianxi Huang, Zhouwu Shu

**Affiliations:** 1 Department of Cardiology, The First Affiliated Hospital of Shantou University Medical College, No.57, Changping Road, Shantou, Guangdong China; 2 Laboratory of Molecular Cardiology, The First Affiliated Hospital of Shantou University Medical College, No.57, Changping Road, Shantou, Guangdong China; 3 Department of Cardiology, Jie Xi’s People Hospital, Jieyang, Guangdong China

**Keywords:** Gut microbiota, Inflammatory proteins, Sleep apnea(SA), Proteomics, Bidirectional mendelian randomization (MR), Phenome-wide association study (PheWAS)

## Abstract

**Background:**

Sleep apnea (SA) is linked to various diseases. This study examines the causal link between the gut microbiome and SA, exploring potential predictive factors and target proteins using a multi-omics approach with a Phenome-wide association study (PheWAS).

**Methods:**

Bidirectional Mendelian Randomization (MR) and Linkage Disequilibrium Score Regression (LDSC) were used to assess the genetic correlation and causal relationships between the gut microbiome and SA. Mediation analysis identified intermediate relationships involving “gut microbiome-inflammatory proteins-SA.” Two-sample MR and colocalization analysis in the deCODE and UK Biobank Pharma Proteomics Project (UKB-PPP) databases identified protein quantitative trait loci (pQTL) associated with SA. Validation analysis used Fenland proteins, methylation quantitative trait loci (mQTL), and expression quantitative trait loci (eQTL). PheWAS screened 29 SA-associated SNPs and matched control SNPs (4:1 ratio) from UK Biobank data chosen through MR and LDSC analyses.

**Results:**

Inverse-variance weighted (IVW) bidirectional MR analysis did not establish a causal link between the gut microbiome and SA. C–C motif chemokine 28 showed causal relationships in both directions (forward IVW, *P* = 0.0336; reverse IVW, *P* = 0.0336). Intermediate connections were found between the Holdemanella genus and urinary plasminogen activator levels with SA. **TIMP4** protein had a significant causal relationship with SA(IVW method: *P* > 0.05, PH4 = 96.1%; *P* = 7.85 × 10^−6^, PH4 in deCODE = 97.4%). **PRIM1** and **BMP8 A** were identified as potential influencers of SA through mQTL and eQTL analyses. PheWAS suggested body impedance and predicted mass as potential predictors of SA.

**Conclusion:**

Bidirectional causal relationships exist between SA and inflammatory proteins, with **TIMP4** identified as a pathogenic factor and potential therapeutic target. **PRIM1** and **BMP8 A** may impact SA risk. Body impedance and predicted mass predict SA significantly.

**Supplementary Information:**

The online version contains supplementary material available at 10.1186/s12986-025-00925-0.

## Introduction

Sleep apnea (SA) is a disorder characterized by respiratory cessation during sleep, leading to frequent awakenings and intermittent hypoxia [1, 2]. SA encompasses obstructive SA (OSA) and central SA (CSA) [3, 4], with OSA being the more prevalent condition to date [4, 5], and OSA being more severe than CSA [3]. Therefore, we will focus on introducing OSA and emphasize our research on it. OSA, a complex disorder, involves various mechanisms and risk factors. These factors comprise craniofacial structural differences, reduced upper airway width, elevated body mass index (BMI), and impaired pharyngeal dilator muscle function, all contributing to upper airway collapse and subsequent episodes of apnea and hypopnea [6–9]. OSA is one of the most prevalent chronic respiratory disorders [10], with a high global incidence [7]. SA is associated with heightened fatigue, including mental and physical fatigue, as well as increased risk of automobile accidents, decreased psychological wellbeing, and diminished overall quality of life [11]. Moreover, SA is linked to stroke [12] and elevated blood levels of reactive oxygen species [13].

Gut microbiota, the communities of bacteria, viruses, fungi, etc., interact with the host [14]. Disturbances in the gut microbiota can result in various disorders, including respiratory [15], circulatory [16], urinary [17], reproductive [18], endocrine [19], hematological [20], musculoskeletal [21], immune [22], digestive [23] and neurological disorders [24]. Similarly, many researches indicate that the gut microbiota has an impact on OSA [25–27]. Reliable studies suggest that dysbiosis of the gut microbiota can generate metabolites and impact the occurrence of SA through the release of inflammatory factors or proteins. Simultaneously, the gut dysbiosis induced by SA may serve as a pathological factor resulting from hypoxia, thereby affecting the abundance of oxygen-sensitive bacteria and contributing to conditions of gastrointestinal inflammation and intestinal permeability(4). Regarding inflammatory proteins, a study has indicated a significant increase in concentrations of pro-inflammatory cytokines such as IL-6 and TNF-a in patients with OSA [28]. For CSA, although the airway is not obstructed, disturbances in the central nervous system can lead to its occurrence. Due to the existence of the gut-brain axis, the gut microbiota can influence the central nervous system through metabolites, neurotransmitters, immune cells, and inflammatory factors [4]. Thus, one possible mechanism is that the gut microbiota influences the occurrence and progression of SA by regulating inflammatory proteins [29, 30].

Mendelian randomization (MR) studies have recently gained widespread application in the field of disease etiology research. In the absence of randomized controlled trials, MR provides a compelling strategy to explore causal relationships between exposure and outcomes. MR employs exposure-relevant single nucleotide polymorphisms (SNPs) as instrumental variables (IVs) to assess the causal impact of genetic proxies for exposure on outcomes [31]. Precisely, this IVs substitution approach mimics a randomized controlled trial (RCT) as SNPs are randomly allocated to offspring during conception, which largely avoids confounding factors as gender and age are unlikely to introduce bias in causal effects [32, 33]. Similarly, reverse causality induced by MR studies is also unlikely as genotype formation occurs prior to the onset of the disease [33, 34]. The linkage disequilibrium score regression (LDSC) enables the assessment of genetic correlation while being unaffected by sample overlap [35]. In this study, we will utilize MR and LDSC analyses to investigate the relationship between gut microbiota, inflammatory proteins, and SA. Additionally, we will employ MR methodology to study protein quantitative trait loci (pQTL) related to SA while also conducting validation analyses using expression quantitative trait loci (eQTL) and methylation quantitative trait loci (mQTL).

Phenome-wide association study (PheWAS) is an approach that analyzes the associations between a collection of phenotypes and specific genetic variations. In recent years, it has become feasible through the integration of electronic health records (EHR) data with large-scale genomic datasets [36–38]. PheWAS fundamentally disrupts the classical genome-wide association study (GWAS) approach, which focuses on testing the associations between a large number of phenotypes (as opposed to a single disease or trait) and individual genetic variations (instead of the millions of variations distributed throughout the entire genome) [39]. Consequently, it is often referred to as reverse GWAS. Expanding the phenotype list and conducting PheWAS on known disease-associated SNP allows for the identification of potential comorbid conditions [40] and traits that may mediate the SNP-disease associations [41]. In our study, we will employ the identification of risk alleles associated with SA to generate a candidate empirical list of SNP and test their pleiotropic associations with the studied disease [42]. Finally, we will validate the obtained results using MR and LDSC analysis methods.

## Materials and methods

**Fig. 1:**
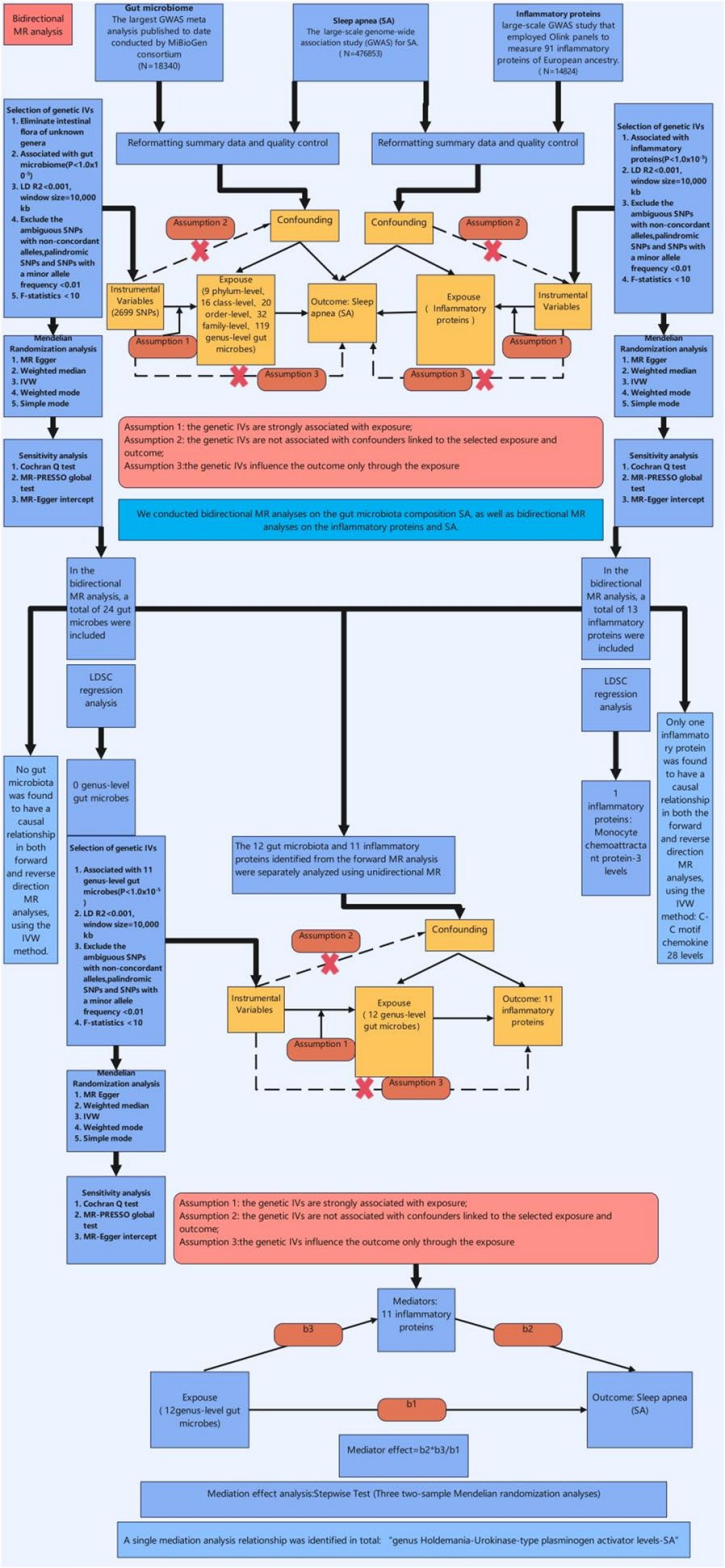
Flowchart illustrating the MR analysis of gut microbiota, inflammatory proteins and SA. SA: Sleep apnea; MR: Mendelian Randomization

**Fig. 2:**
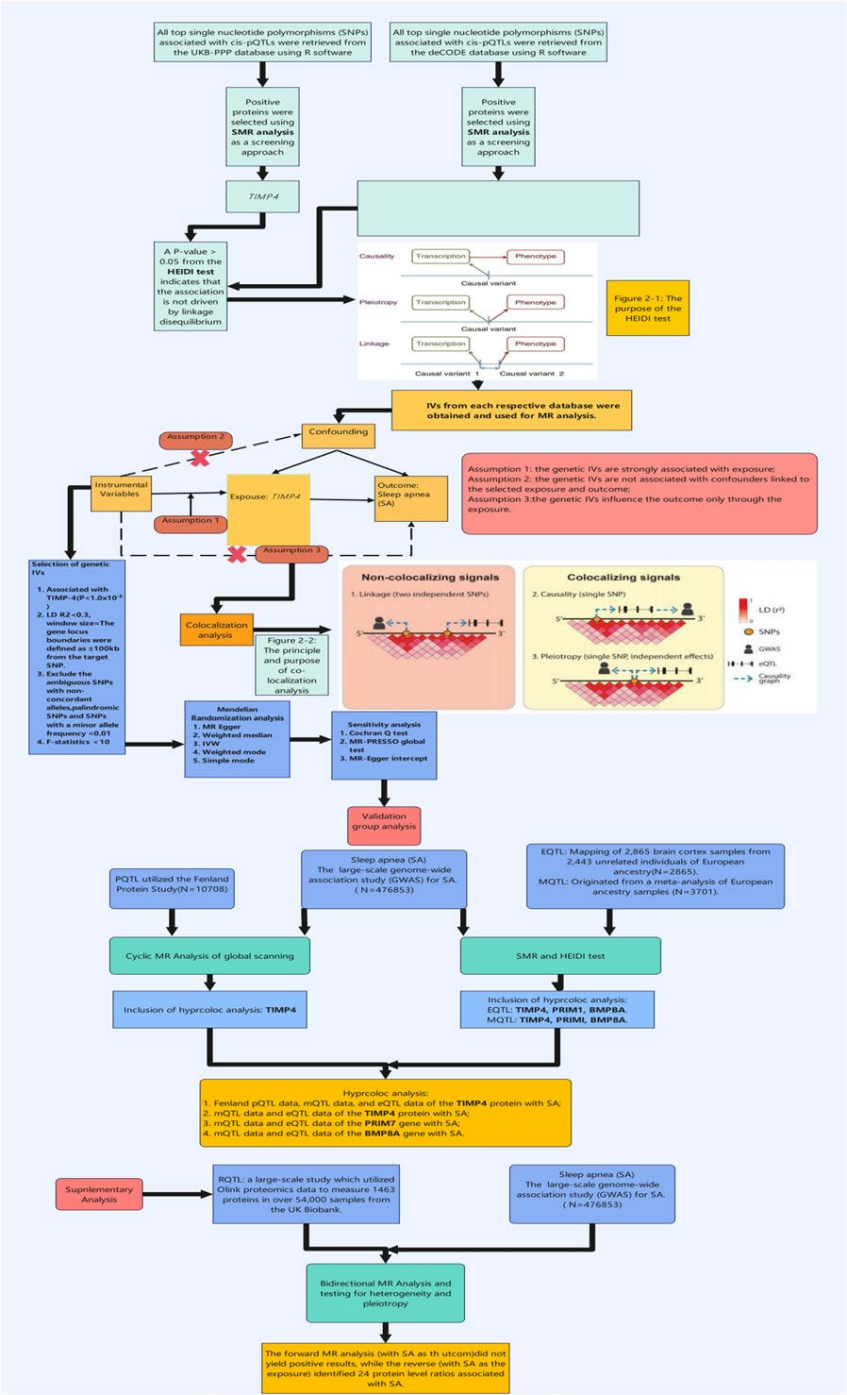
Flowchart illustrating the pQTL,eQTL, and mQTL analysis of SA. pQTL: protein quantitative trait loci; mQTL: methylation quantitative trait loci; eQTL: expression quantitative trait loci; SA: Sleep apnea

**Fig. 3:**
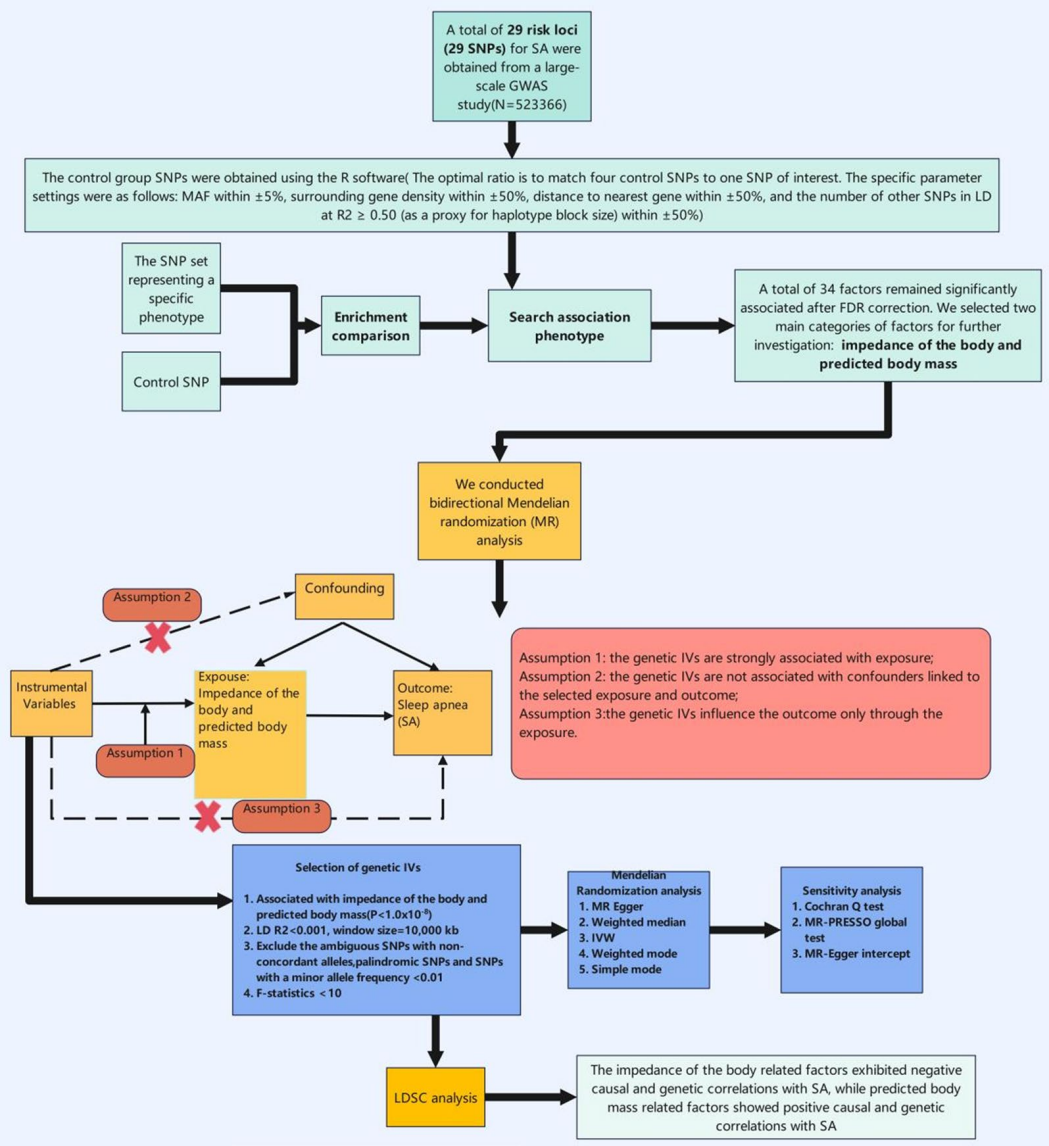
Flowchart illustrating the PheWAS analysis of SA. SA: Sleep apnea; LDSC: Linkage Disequilibrium Score Regression; PheWAS: Phenome-wide association study

*Study design* We conducted LDSC and two-sample MR analyses to estimate the genetic correlations and causal relationships among the gut microbiome, inflammatory proteins, and SA. To enhance the robustness of our results, we implemented bidirectional MR analyses, which were also applied after the PheWAS analyses. LDSC was utilized to support the robustness of MR findings. Additionally, it was essential to conduct pleiotropy and heterogeneity tests, as results that passed these tests were deemed more reliable; if the tests were not passed, we employed a random effects model for MR. To control for bias, we utilized the LDlink (https://ldlink.nih.gov/?tab=home) portal to verify our results, a procedure that was similarly applied to the MR analyses following PheWAS. An overview of the study design is illustrated in Fig. 1. Furthermore, we performed summary data-based MR (SMR) analysis and HEIDI testing for SA using the UK Biobank Pharma Proteomics Project (UKB-PPP) and deCODE databases, alongside MR and colocalization analyses. We conducted validation analyses using Fenland protein, mQTL, and eQTL data. These analyses did not require pleiotropy and heterogeneity assessments or bias checks, as in most cases, the effective IV consisted of a single top SNP. Its flow chart is shown in Fig. 2. Finally, we conducted PheWAS experiments and validated the results using two-sample MR and LDSC analyses, as shown in Fig. 3. All analyses were performed using R software (version 4.3.1) and the SMR computational application (version 1.3.1).

### Data source

#### GWAS datasets for gut microbes, inflammatory proteins, and SA

The gut microbiome data associated with this study were obtained from the most significant GWAS meta-analysis conducted by the MiBioGen Consortium (https://mibiogen.gcc.rug.nl/menu/main/home/) [43]. This study harmonized 16S rRNA gene sequencing profiles and genotype data from 24 cohorts. The majority of participants had European ancestry (N = 13,266).

The data for inflammatory proteins were derived from a large-scale GWAS study that employed Olink panels to measure 91 inflammatory proteins in 14,824 plasma samples of European ancestry from 11 cohorts (GWAS summary statistics are available for download at https://www.phpc.cam.ac.uk/ceu/proteins/ and the EBI GWAS Catalog https://www.ebi.ac.uk/gwas/(accession GCST90270765-GCST90270855)) [44].

Our SA data (Online data: https://gwas.mrcieu.ac.uk/datasets/ebi-a-GCST90018916/. Local data: https://ftp.ebi.ac.uk/pub/databases/gwas/summary_statistics/GCST90018001-GCST90019000/GCST90018916/) were derived from a large-scale meta-analysis study conducted in the Japan Biobank (N = 179,000), which encompassed 220 deep phenotyping GWASs for diseases, biomarkers and drug usage [45]. This study integrated past medical history and electronic health record text mining. Through a meta-analysis of the UK Biobank and FinnGen datasets (n_total_ = 628,000), approximately 5,000 novel loci were identified [45]. In our study, we only used data from the European population.

### PheWAS datasets

The data for PheWAS analysis were sourced from the UK Biobank. This database aggregates information on 19,586 different phenotypes, including diagnoses, family history, lifestyle factors, current health status, anthropometric measurements, physiological traits, treatment records, biochemical analyses, and mental health [46]. The sources, original questionnaires, and measurement details for these phenotypes can be obtained from the official website of the UK Biobank (https://www.nealelab.is/uk-biobank/).

The body impedance and body mass prediction online data used for MR analysis following PheWAS were obtained from another study involving a European population (N = 454,840). The relevant links to the online data are provided in Supplementary Table S1. Data used for LDSC analysis, including body impedance and body mass prediction following PheWAS, were obtained from one study (n = 174,489) utilizing the UK Biobank dataset [47]. In this study, the researchers discovered 6,157 statistically significant associations between 247 diseases and 109 RFPRS [47]. The relevant links to the local data are provided in Supplementary Table S2.

### PQTL datasets

The UKB-PPP (https://www.synapse.org/#!Synapse:syn51365301) is a competitive pre-competitive consortium composed of 13 biopharmaceutical companies that delineate the plasma proteomic characteristics of 54,219 UK Biobank participants [48]. The consortium conducted comprehensive pQTL mapping for 2,923 proteins, identifying 14,287 primary genetic associations, 81% of which were previously undescribed, as well as ancestry-specific pQTL mapping for non-European individuals [48].

The deCODE (https://www.decode.com/summarydata/) database describes GWASs of plasma protein levels determined by 4,907 aptamer assays in 35,559 Icelanders. The study revealed 18,084 associations between sequence variants and plasma protein levels (pQTLs), with 19% being rare variants (minor allele frequency, MAF < 1%) [49]. The research explored associations between plasma protein levels and 373 diseases and other traits, identifying 257,490 associations [49].

### Validation group datasets

Our validation set for pQTL utilized the Fenland Protein Study (http://www.omicscience.org/apps/pgwas). In this study, a cohort of 10,708 individuals of European ancestry underwent plasma proteome profiling, measuring the plasma abundance of 4,775 distinct protein targets and 10.2 million genetic variants using a standardized workflow [50]. Our eQTL data was derived from eQTL mapping of 2,865 brain cortex samples from 2,443 unrelated individuals of European ancestry [51]. In contrast, our mQTL data originated from a meta-analysis of European ancestry samples (N = 3701) conducted by Hatton et al. Both datasets are available for download from the YangLab website (https://yanglab.westlake.edu.cn/software/smr/#DataResource).

*Genetic IVs: (1) a*. For the IVs required for MR analysis on the gut microbiome, inflammatory proteins, and SA: Previous studies have shown that the number of IVs meeting the criteria is very low when using the genome-wide significance threshold (*p* < 5 × 10^−8^) [43, 52–55]. Therefore, from an evidential perspective, a relatively less stringent threshold (*p* < 1 × 10^−5^) [43, 55] can capture potential strong associations and yield more comprehensive results.. In the analysis, for gut microbiota, when *P* < 5 × 10^−8^, we can identify only 27 SNPs, whereas when *P* < 1 × 10^−5^, we can identify 2699 SNPs; for inflammatory proteins, when *P* < 5 × 10^−8^, we can identify 327 SNPs, while when *P* < 1 × 10^−5^, we can identify 1820 SNPs. Using P < 5 × 10^−8^ not only reduces the number of SNPs but also results in the absence of certain gut microbiota and inflammatory protein categories. Therefore, considering the consistency of our mediation analysis chain, when SA is the final outcome variable, we continue to uniformly choose *P* < 1 × 10^−5^ as our threshold. **b.** For the IVs required for MR analysis on the body impedance and body mass obtained after PheWAS with SA: In the forward analysis (with SA as the exposure factor, p < 5 × 10^−8^), we found only one SNP (rs11075985). We queried this SNP at the National Center for Biotechnology Information (NCBI) (https://www.ncbi.nlm.nih.gov/snp/rs11075985) and found no reported clinical significance. Based on previous experience [56], we used a more liberal threshold (*p* < 5 × 10^−5^). However, in the reverse MR analysis (with SA as the outcome factor), we applied a strict threshold (*p* < 5 × 10^−8^), and there were still sufficient SNPs available. **c.** For our proteomic analyses, in the pQTL, mQTL, and eQTL analyses, we continued to employ a stringent threshold (*p* < 5 × 10^−8^) due to the availability of a sufficient number of SNPs. **(2) a.** We performed clustering (R^2^ < 0.001, window size = 10,000 kb) to exclude variants in strong linkage disequilibrium (LD) and ensure the independence of each SNP. **b.** For the colocalization analysis in pQTL, mQTL, and eQTL, we set the R^2^ threshold to 0.3 and defined the range around the genetic variants of the corresponding proteins as ± 100 kb [57]. **(3)** We excluded SNPs with minor allele frequency (MAF) < 0.01, SNPs with ambiguous alleles, and palindromic SNPs. **(4)** To account for pleiotropy introduced by instrumental variables, we calculated the r^2^ and the F-statistic for each SNP [58, 59]. The computation for R^2^ and the F-statistic is as follows:

$$\begin{array}{l}{R}^{2}=\frac{2*{\beta }^{2}*EAF*\left(1-EAF\right)}{\left[2*{\beta }^{2}*EAF*\left(1-EAF\right)+2*{\left(se\left(\beta \right)\right)}^{2}*N*EAF*\left(1-EAF\right)\right]}\\ F=\frac{N-k-1}{k}*\frac{{R}^{2}}{1-{R}^{2}}\end{array}$$

Among these formulas, β represents the effect size of the genetic variant of interest; EAF represents the effect allele frequency of the genetic variant of interest; se (β) represents the standard error of the effect size of the genetic variant of interest; R^2^ represents the proportion of exposure variance explained by the IVs (determinant coefficient of the regression equation); N represents the sample size; and k represents the number of SNPs. SNPs with an F-statistic of less than 10 are defined as weak instruments and thus are removed [59].

### Statistical analysis

#### Analysis of gut microbiome, inflammatory proteins, and SA:

*MR analysis* A compelling MR study should adhere to three assumptions: (1) a robust and strong correlation between instrumental variables and the exposure factor; (2) independence between instrumental variables influencing the “exposure-outcome” relationship and confounding factors; (3) genetic variation can only affect the occurrence of the outcome through the exposure factor and not through any other pathways [60]. In our study, we investigated the causal relationship between gut microbiota and SA, as well as the causal effect of inflammatory proteins on SA, using bidirectional MR analysis and employing five methods: inverse variance weighted (IVW), MR Egger, weighted median (WM), weighted mode, and simple mode. After identifying gut microbiota and inflammatory proteins that showed statistical significance in relation to SA, we further analyzed the causal relationship between gut microbiota and inflammatory proteins using the five methods mentioned above (with gut microbiota as the exposure). IVW was the preferred method when all selected SNPs served as valid IVs [34, 61]. MR Egger and WM methods served as complementary analyses, providing robust estimates under relaxed assumptions. WM allowed for the inclusion of up to 50% invalid SNPs, while MR Egger detected horizontal pleiotropy and heterogeneity across all SNPs, particularly in the presence of evidence suggesting horizontal pleiotropy [61, 62]. P-values were adjusted considering potential correlations between different phenotypes, considering that the Bonferroni threshold might be overly conservative; therefore, we employed false discovery rate (FDR) correction [63].

Sensitivity analyses Sensitivity analyses were conducted to examine potential violations of horizontal pleiotropy and heterogeneity in the MR estimates. Horizontal pleiotropy was detected using the MR Egger method [64], considering *p* < 0.05 as evidence of pleiotropy. The negative result of the pleiotropy test indicates that instrumental variables may be effective and not influenced by pleiotropy effects. Heterogeneity was assessed using Cochran’s Q test, with *P* < 0.05 indicating evidence of heterogeneity. Additionally, we utilized the MR-PRESSO method to assess outlier effects and potential horizontal pleiotropy [65], enabling the identification and removal of outliers based on this approach.

*Mediation analysis* Based on the analyses above, we established an intermediate analysis chain, commonly known as causal stepwise testing or two-step [66] method, involving the gut microbiota, inflammatory proteins, and SA. Finally, we used the coefficient product method [66] in R software (R-4.3.1) to compute the mediation effect and draw corresponding conclusions.

*Genetic correlation analysis* Based on the results obtained from MR analysis, we used LDSC to estimate the genetic correlation (r_g_) between the selected gut microbiota and SA, as well as between the selected inflammatory proteins and SA. LDSC allows for the quantitative analysis of the contribution of different components of the inflation test statistic by examining the association between the test statistic and linkage disequilibrium [67]. It provides valuable insights into the genetic correlation between complex traits and diseases, aiding in the prioritization of potential causal relationships [35]. The computation method is as follows:

$${r}_{g}:={\rho }_{g}/\sqrt{{h}_{1}^{2}{h}_{2}^{2}}$$

Genetic covariance (ρ_g_) can be estimated using LDSC on the product of the z-scores of variations in Trait 1 and Trait 2, denoted as z1j and z2j, representing the LD regression slope of the LD score [68]. The genetic correlation (r_g_) is obtained by standardizing the genetic covariance using the SNP heritability (hi^2^, representing the heritability of SNP in study i) [35].

### PQTLs analysis

*SMR and HEIDI Test* We initially obtained the top-snp with the lowest P-value for cis-pQTLs from UKB-PPP and deCODE databases [48, 49] (Detailed data of the screening can be found in Supplementary Table S3 and Table S4). Subsequently, we employed summary data-based Mendelian randomization (SMR) analysis to identify proteins associated with SA. SMR is an analytical method that combines summary data from GWAS with expression quantitative trait loci (eQTL) to identify genes whose expression levels are related to complex traits due to pleiotropy [69]. Here, we utilize this approach for pQTL analysis. To evaluate the possibility of linkage effects influencing our results, we performed heterogeneity in dependent instruments (HEIDI) tests. We used the HEIDI method to test for heterogeneity in the b_xy_ values estimated for multiple SNPs in the pQTL region. A HEIDI test p-value > 0.05 indicates that the association is not driven by linkage disequilibrium [69]. Finally, we applied FDR correction to our results. All the analyses, as mentioned earlier, were performed using the R software (R-4.3.1).

*MR and colocalization analysis* We subsequently performed a two-sample MR analysis on the protein (**TIMP4*)*** selected using the method mentioned earlier and their association with SA. We then conducted a colocalization analysis of the results. Colocalization analysis, based on Bayesian principles, is an essential post-GWAS approach aimed at identifying genetic variants associated with a phenotype based on GWAS results [70]. This method involves four hypotheses and based on these hypotheses, the higher the statistical probability of hypothesis H4 (PH4), the better it explains how significant signal loci affect the phenotype. Some studies consider associations with PH4 > 0.8 to have strong colocalization support, while associations with 0.8 > PH4 > 0.5 are considered to have moderate colocalization support [71, 72]. Subsequently, heterogeneity and pleiotropy tests, as well as MR-PRESSO tests, were performed.

*Validation group* We utilized proteins from the Fenland cohort to validate the results obtained from the two proteomic database analyses. After FDR correction, in the UKB-PPP database, the SMR and HEIDI tests yielded positive results, while in the deCODE database, MR and colocalization analysis produced positive results. Additionally, we attempted validation from a non-proteomic perspective using mQTL and eQTL data. In the context of Fenland proteomics, a global MR scan was conducted on the 4,979 plasma proteins obtained from this cohort, followed by FDR correction, heterogeneity, and pleiotropy analyses. For the mqtl and eqtl analyses, we employed the smr.exe software for the overall SMR analysis and HEIDI test. Finally, a hypothesis prioritization analysis for multi-trait colocalization (hyprcoloc) was performed on the **TIMP4**-related Fenland protein data, eQTLdata, mQTL data, and SA data. This test is a Bayesian method that utilizes a novel branch and constrained cluster splitting algorithm to identify subsets of traits (which we refer to as clusters) showing colocalization on different causal variants within a genetic locus [73]. Furthermore, for the positive results obtained from the overall SMR analysis of mQTL and eQTL data, we conducted hyprcoloc analysis in an attempt to identify other genes associated with SA.

### PheWAS analysis

*Selection of SA risk alleles* We obtained 29 risk loci strongly associated with SA from a large-scale GWAS study (see Supplementary Table S5). The study conducted a GWAS of SA in five cohorts (N_Total_ = 523,366) and discovered 29 variants in the 23andMe GWAS after adjusting for BMI [74].

*Control SNP set* We developed a control set of SNPs using the R software (R-4.3.1), which consisted of a randomly sampled set of SNPs with similar allele frequencies, LD patterns, distance to nearest genes, and gene density as the SNPs under study (see Supplementary Table S6). Previous studies have suggested that the optimal ratio is to match four control SNPs to one SNP of interest [75]. The specific parameter settings were as follows: MAF within ± 5%, surrounding gene density within ± 50%, distance to nearest gene within ± 50%, and the number of other SNPs in LD at R2 ≥ 0.50 (as a proxy for haplotype block size) within ± 50% [39].

*Search for phenotypic associations based on gene enrichment* The principles for phenotype selection are as follows: **(1)** Exclude phecode with no less than 200 cases [76]. **(2)** Binary phenotypes with fewer than 100 cases and continuous and categorical ordered phenotypes with sample sizes of fewer than 10,000 were excluded [77]. **(3)** International Classification of Diseases (ICD)-coded binary phenotypes without principal ICD codes or with external causes (codes as Z00–Z99) were also excluded [78]. In general, in gene enrichment analysis, if a set of SNPs representing a phenotype 1 of interest (SA) shows significant enrichment compared to control SNPs for another phenotype 2, it can be inferred that the phenotype 1 of interest (SA) may be associated with phenotype 2 [39]. Finally, we performed FDR correction on the p-values of our results. The final results (A total of 2502 phenotype outcomes were selected after screening) can be found in Supplementary Table S7.

*MR and LDSC analysis* We searched for phenotypes with significant statistical significance after FDR correction in the outcomes. We identified two major categories of factors that showed significant associations with our phenotype of interest, SA: body impedance and body predicted mass. Subsequently, we performed bidirectional MR analysis and LDSC analysis on a total of 10 subcategories (Impedance of arm (left), impedance of arm (right), impedance of leg (left), impedance of leg (right), impedance of whole body, arm predicted mass (left), arm predicted mass (right), leg predicted mass (left), leg predicted mass (right), trunk predicted mass) within these two major categories of factors. Heterogeneity and pleiotropy tests, as well as MR-PRESSO tests, were conducted as a final step in our analysis.

## Results

### Analysis of gut microbiome, inflammatory proteins, and SA

**Fig. 4:**
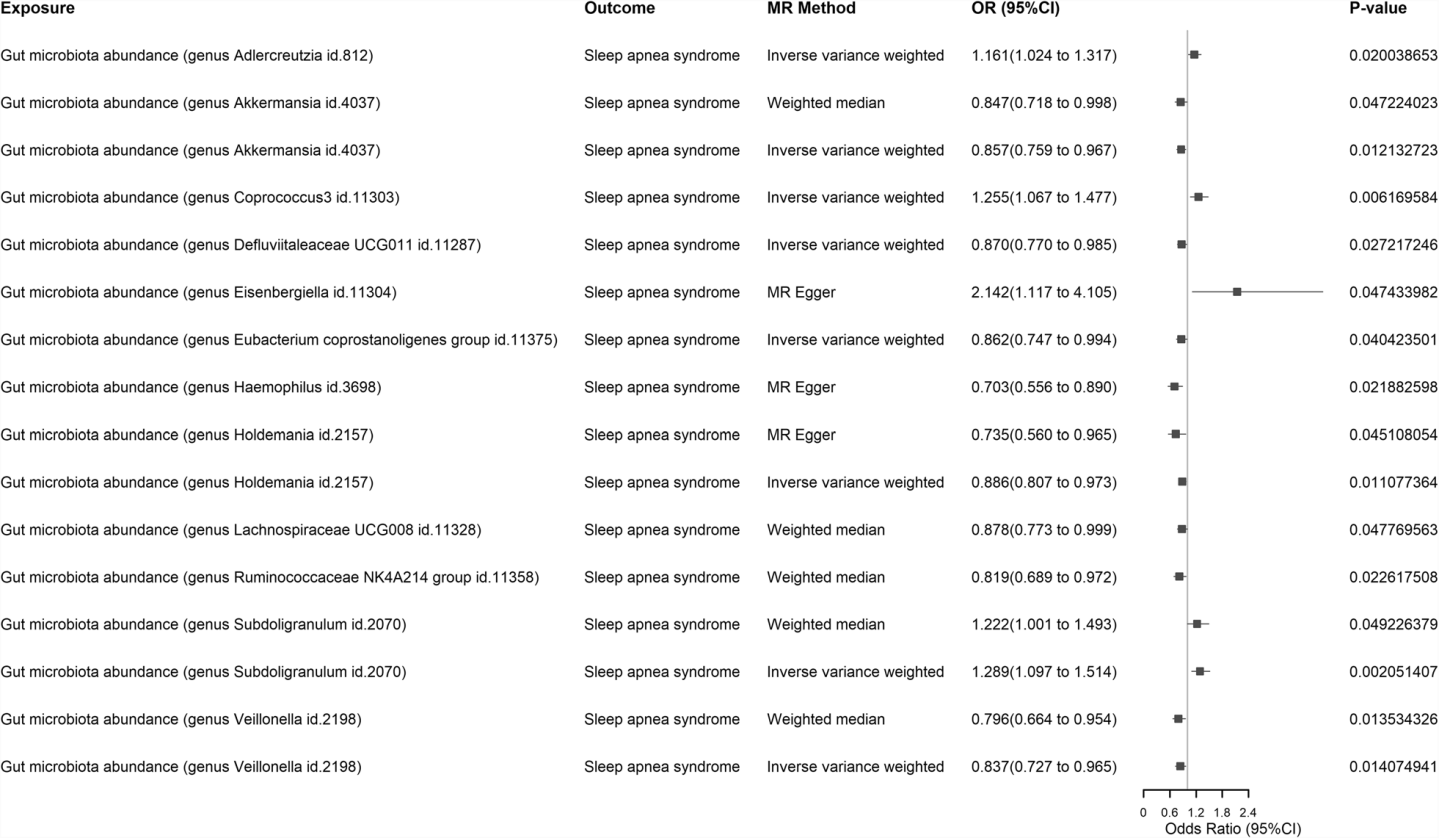
Forest maps of 12 genus-level gut microbes in positive MR Analysis with significant causal relationship to SA in at least one MR Method. Exposure: Signifies our exposure factor as gut microbiota. Outcome: Indicates our outcome factor as sleep apnea. MR Method: Denotes the analytical method employed in our study. OR (95% CI): Represents the odds ratio and its corresponding 95% confidence interval. P-value: Signifies the p-value obtained from the respective method of analysis. MR: Mendelian Randomization; SA: Sleep apnea

**Fig. 5:**
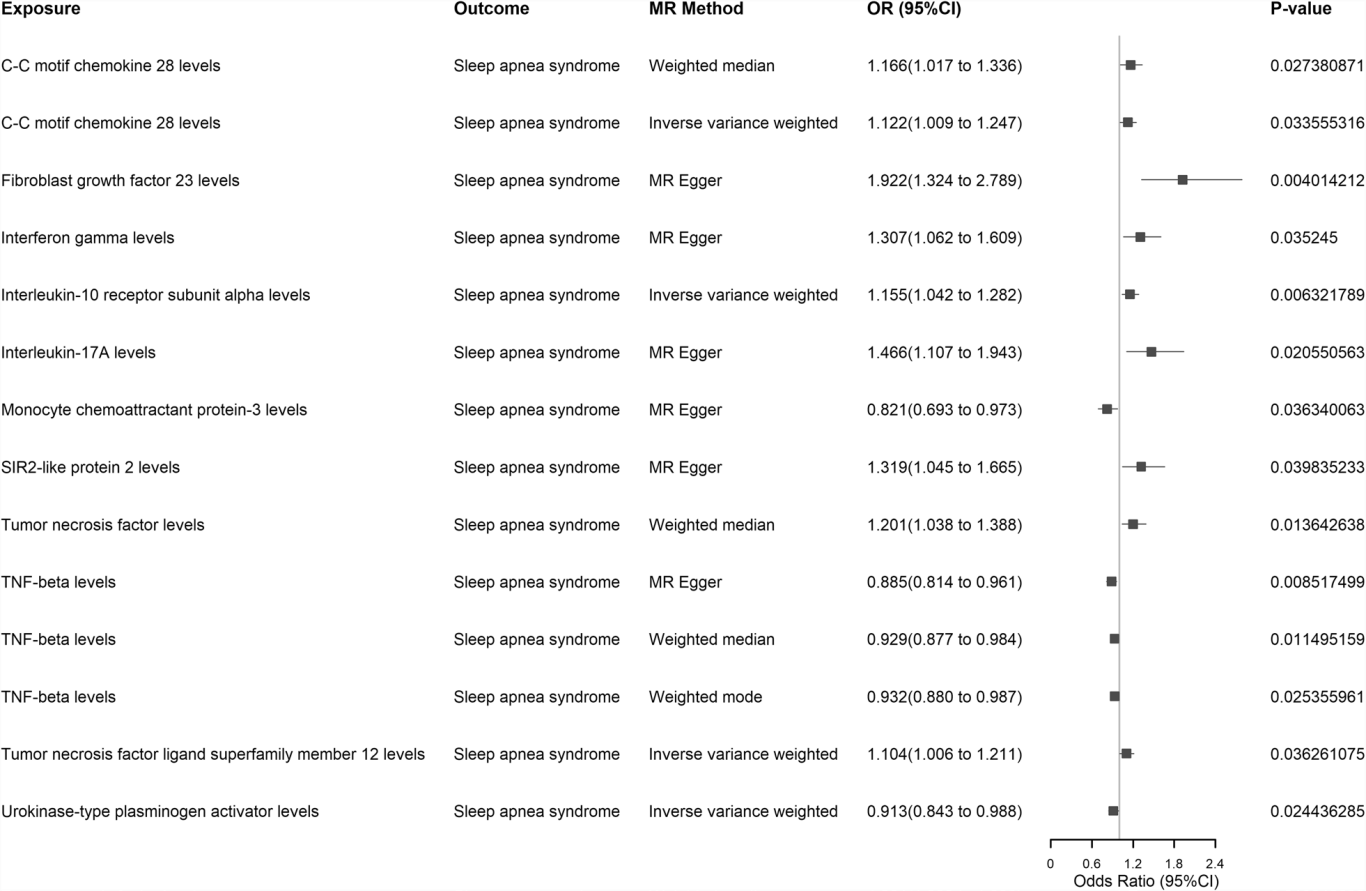
Forest map of 11 inflammatory proteins in positive MR Analysis with a significant causal relationship with SA in at least one MR Method. Exposure: Signifies our exposure factor as inflammatory proteins. Outcome: Indicates our outcome factor as sleep apnea. MR Method: Denotes the analytical method employed in our study. OR (95% CI): Represents the odds ratio and its corresponding 95% confidence interval. P-value: Signifies the p-value obtained from the respective method of analysis. SA: Sleep apnea; MR: Mendelian Randomization

*MR Analysis* As some intestinal microbiota belong to unknown species, we excluded them, including 3 family-level and 12 genus-level. We initially obtained all valid IVs for gut microbiota (including 9 phylum-level, 16 class-level, 20 order-level, 32 family-level, and 119 genus-level gut microbes, as detailed in Supplementary Table S8). In the forward MR analysis (with gut microbiota as the exposure), we found that 12 genus-level gut microbes had significant causal relationships with SA in at least one MR method (see Fig. 4 for forest plots and Supplementary Figure for circular heatmaps), which remained significant after FDR correction (details in Supplementary Table S9). Additionally, we identified 11 inflammatory proteins (with inflammatory proteins as the exposure) that showed causal relationships with SA in at least one MR method (see Fig. 5 for forest plots), which also remained statistically significant after FDR correction (details in Supplementary Table S10). In our reverse MR analysis (with SA as the exposure), we identified another 13 genus-level gut microbes (see Supplementary Table S11) and 5 inflammatory proteins (see Supplementary Table S12) that remained significantly associated with SA after FDR correction, as depicted in the forest plots provided in the Supplementary Fig. 2 and Supplementary Fig. 3. Furthermore, we discovered that the genus Lachnospiraceae UCG008 showed causal relationships with SA in both forward and reverse MR analysis (forward WM method, *P* = 0.0478, OR = 0.8785, 95% CI: 0.7727–0.9987; reverse WM method, *P* = 0.0244, OR = 1.1807, 95% CI: 1.0217–1.3644). Additionally, we identified three inflammatory proteins that exhibited causal effects with SA in both forward and reverse MR analysis, namely C–C motif chemokine 28 levels (forward IVW method, *P* = 0.0336, OR = 1.1216, 95% CI: 1.0090–1.2468; reverse IVW method, *P* = 0.0336, OR = 0.9459, 95% CI: 0.8986–0.9957), Fibroblast growth factor 23 levels (forward MR Egger method, *P* = 0.0040, OR = 1.9217, 95% CI: 1.3239–2.7893; reverse WM method, *P* = 0.0456, OR = 1.0872, 95% CI: 1.0016–1.1802) and Tumor necrosis factor ligand superfamily member 12 levels (forward IVW method, *P* = 0.0362, OR = 1.1040, 95% CI: 1.0064–1.2111; reverse MR Egger method, *P* = 0.0312, OR = 0.8403, 95% CI: 0.7206–0.9799). Notably, among these, only C–C motif chemokine 28 levels exhibited significant causal relationships with SA in both directions of the bidirectional MR analysis, including the IVW method. In our MR analysis between gut microbiota and inflammatory proteins (with gut microbiota as the exposure), we identified a causal effect of genus Holdemania on Urokinase-type plasminogen activator levels (IVW method, *P* = 0.0082, OR = 1.1163, 95% CI: 1.0289–1.2111), which remained statistically significant after FDR correction (see Supplementary Table S13).

*Sensitivity analyses* We conducted heterogeneity and pleiotropy tests for the bidirectional MR analysis between gut microbiota and SA (heterogeneity and pleiotropy results for forward MR analysis are shown in Supplementary Tables S14 and S15 and for reverse MR analysis in Supplementary Tables S16 and S17), as well as for the unidirectional MR analysis between inflammatory proteins and SA (The heterogeneity and pleiotropy results of forward MR Analysis were shown in Supplementary Table S18 and S19, the heterogeneity and pleiotropy results of reverse MR Analysis were shown in Supplementary Table S20 and S21, and the heterogeneity and pleiotropy results of gut microbiome and inflammatory protein were shown in Supplementary Table S22 and S23). The results of these analyses did not provide evidence of significant heterogeneity or pleiotropy. Additionally, we performed MR-PRESSO tests for the forward analysis of gut microbiota and SA (shown in Supplementary Table S24), inflammatory proteins and SA (shown in Supplementary Table S25), and gut microbiota and inflammatory proteins (shown in Supplementary Table S26), and no significant outliers were detected.

*Mediation analysis* Through the MR mentioned above analysis, we aimed to identify a potential mediation pathway of “gut microbiota-inflammatory proteins-SA.” We found a causal relationship between genus Holdemania and SA (IVW method, *P* = 0.0110, b = − 0.1207, 95% CI: − 0.2138 to − 0.0275), a causal relationship between Urokinase-type plasminogen activator levels and SA (IVW method, *P* = 0.0244, b = − 0.0911, 95% CI: − 0.1704 to − 0.0117) and a causal relationship between genus Holdemania and Urokinase-type plasminogen activator levels (IVW method, *P* = 0.0082, b = 0.1100, 95% CI: 0.0284 to 0.1915). Therefore, we identified the mediation pathway of “genus Holdemania-Urokinase-type plasminogen activator levels-SA” (b_mediator effect_ = 0.0109, 95% CI: 0.0001 to 0.0268). Refer to Supplementary Table S27 for more details.

*Genetic correlation analysis* We conducted LDSC analysis for all gut microbiota and inflammatory proteins identified in both forward and reverse MR analyses with respect to SA. The results were disappointing, as no gut microbial taxa exhibited significant genetic correlations with SA among the identified gut microbiota. At the same time, only one inflammatory protein, Monocyte chemoattractant protein-3 levels, showed a genetic correlation with SA (r_g_ = 0.4265, *P* = 0.0024) among the identified inflammatory proteins. In the LDSC analysis between gut microbiota and inflammatory proteins, we did not observe significant results (r_g_ = − 0.3458, *P* = 0.4935). The LDSC analysis results for gut microbiota, and SA can be found in Supplementary Table S28. In contrast, the LDSC analysis results for inflammatory proteins and SA can be found in Supplementary Table S29. Table 1 presents the summary results of LDSC analyses for the associations between gut microbiota and SA, as well as inflammatory proteins and SA.

**Table 1:** Results of LDSC analysis for gut microbiota and SA, as well as inflammatory proteins and SA

trait1	trait2	r_g_	r_g__p	r_g__se
*Sleep apnea syndrome & Gut microbiota*				
Sleep apnea syndrome	genus.Adlercreutzia.id.812	− 0.3867004	0.4374587	0.4980097
Sleep apnea syndrome	genus.Akkermansia.id.4037	0.08850223	0.6409167	0.189749
Sleep apnea syndrome	genus.Coprococcus3.id.11303	0.1477373	0.6630021	0.3390244
Sleep apnea syndrome	genus.Eisenbergiella.id.11304	0.2487305	0.6203796	0.5021688
Sleep apnea syndrome	genus.Haemophilus.id.3698	− 0.04048243	0.9296855	0.4587738
Sleep apnea syndrome	genus.Holdemania.id.2157	− 0.05594375	0.862702	0.3234947
Sleep apnea syndrome	genus.Subdoligranulum.id.2070	− 0.2119983	0.3086544	0.2082397
Sleep apnea syndrome	genus Defluviitaleaceae UCG011 id.11287	− 0.3307035	0.5209776	0.5152436
Sleep apnea syndrome	genus Eubacterium coprostanoligenes group id.11375	0.02570413	0.905524	0.2165723
Sleep apnea syndrome	genus Ruminococcaceae NK4 A214 group id.11358	− 0.2834546	0.2859558	0.2656469
Sleep apnea syndrome	genus Christensenellaceae R 7 group id.11283	− 0.1303906	0.3924443	0.1524692
Sleep apnea syndrome	genus Erysipelotrichaceae UCG003 id.11384	− 0.08522951	0.6409975	0.1827765
Sleep apnea syndrome	genus Eubacterium brachy group id.11296	− 0.09053118	0.9004682	0.7238442
Sleep apnea syndrome	genus Eubacterium hallii group id.11338	− 0.1990464	0.4560278	0.2670318
*Sleep apnea syndrome & Inflammatory proteins*				
Sleep apnea syndrome	C–C motif chemokine 28 levels	− 0.1472259	0.1719328	0.1077773
Sleep apnea syndrome	Fibroblast growth factor 23 levels	0.12359	0.3010951	0.119516
Sleep apnea syndrome	Interferon gamma levels	0.04503645	0.8537393	0.2442993
Sleep apnea syndrome	Interleukin-10 receptor subunit alpha levels	0.2623333	0.09385009	0.1565768
Sleep apnea syndrome	Interleukin-17 A levels	0.09433837	0.4164577	0.116097
Sleep apnea syndrome	Monocyte chemoattractant protein-3 levels	0.4265154	0.002450668	0.1407936
Sleep apnea syndrome	SIR2-like protein 2 levels	0.2232764	0.3415379	0.2347483
Sleep apnea syndrome	Tumor necrosis factor levels	0.2138342	0.7114973	0.5781711
Sleep apnea syndrome	TNF-beta levels	0.4757732	0.08526321	0.2764626
Sleep apnea syndrome	Tumor necrosis factor ligand superfamily member 12 levels	− 0.09706207	0.5410669	0.1588056
Sleep apnea syndrome	Urokinase-type plasminogen activator levels	0.1461299	0.2475434	0.1263731
Sleep apnea syndrome	Interleukin-4 levels	− 0.2531366	0.09922725	0.1535453
Sleep apnea syndrome	Tumor necrosis factor ligand superfamily member 14 levels	0.2352239	0.1560802	0.1658399

*SA* sleep apnea; *LDSC* linkage disequilibrium score regression; r_g_: genetic correlation from LDSC analysis; r_g__p: p-value of genetic correlation from LDSC analysis; r_g__se: standard error of genetic correlation from LDSC analysis

### PQTLs analysis

**Fig. 6:**
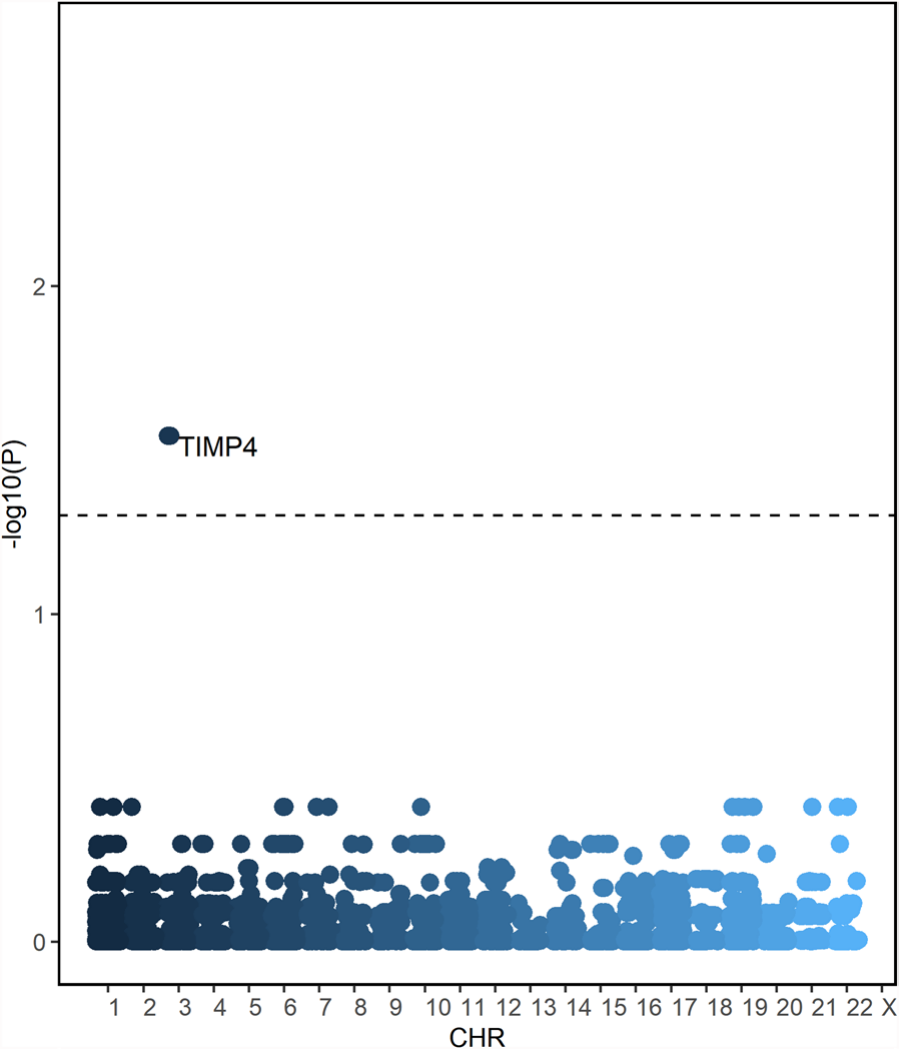
Manhattan map of results of SMR analysis of all proteins with SA in the UKB-PPP database. CHR: represents chromosome. -log10(P): the negative logarithm of the p-value. UKB-PPP: UK Biobank Pharma Proteomics Project; SA: Sleep apnea; SMR: Summary Data-Based Mendelian Randomization

*SMR and HEIDI Test* Initially, we performed SMR analysis on all proteins from the UKB-PPP dataset with respect to SA (see Supplementary Table S30, Manhattan plot in Fig. 6). We identified one proteins that remained significant after FDR correction: **TIMP4** (*P* = 3.05 × 10^−5^, P_adjust_ = 0.0286, top snp: rs167408). However, we observed that **TIMP4** is located on an autosomal chromosome (Chromosome 3: 12,153,068-12,158,912, GRCh38; Chromosome 3: 12,194,568-12,200,412, GRCh37). Subsequently, we performed SMR analysis on all proteins from the deCODE database using the same approach (see Supplementary Table S31). Unfortunately, after FDR correction, we did not find significant associations for **TIMP4** (*P* = 3.48 × 10^−5^, P_adjust_ = 0.0882, top snp: rs184262), but we referred to this as a potential causal relationship [79] and retained it for further analysis. In performing the HEIDI test on the protein **TIMP4** in the deCODE and UKB-PPP datasets, we observed that the P-values were all greater than 0.05 (*P* = 0.3807, deCODE dataset; *P* = 0.5461, UKB-PPP dataset), indicating that this association is not driven by linkage disequilibrium.

**Fig. 7:**
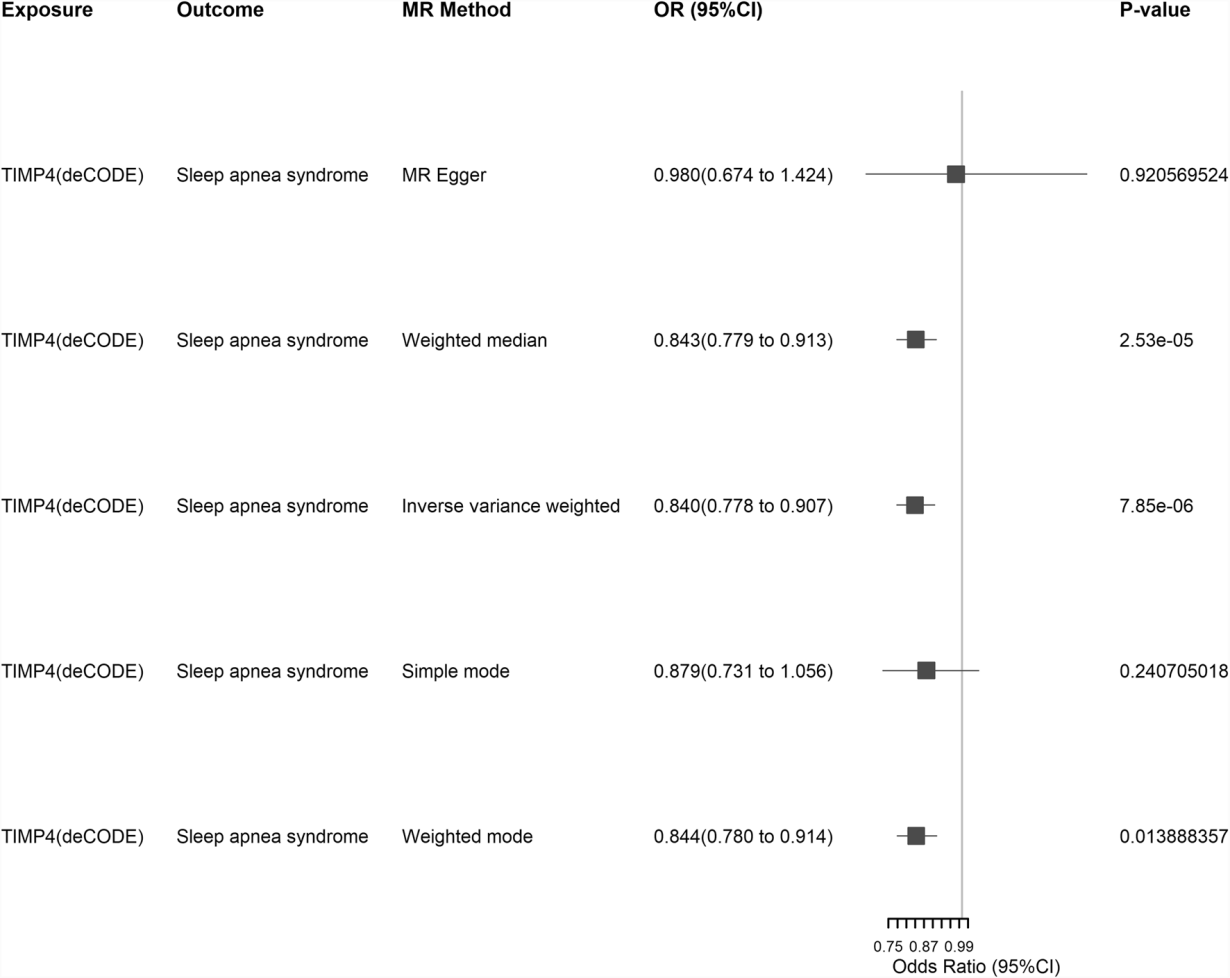
Forest map of the results of MR Analysis of **TIMP4** and SA in the deCEDE database. Exposure: Signifies our exposure factor as **TIMP4**. Outcome: Indicates our outcome factor as sleep apnea. MR Method: Denotes the analytical method employed in our study. OR (95% CI): Represents the odds ratio and its corresponding 95% confidence interval. P-value: Signifies the p-value obtained from the respective method of analysis. SA: Sleep apnea; MR: Mendelian Randomization

*MR and Colocalization Analysis* We first obtained IVs for the **TIMP4** gene region (Chromosome 3: 12,153,068-12,158,912) from the UKB-PPP and deCODE databases, respectively (specific data can be found in Supplementary Table S32 and Table S33). During the screening of IVs in the UKB-PPP database, we identified a total of four IVs, but no meaningful results were obtained in the MR analysis. Subsequently, our MR analysis revealed a significant causal relationship between **TIMP4** and SA (deCODE, IVW method, P = 7.85 × 10^−6^, OR = 0.8399, 95% CI: 0.7780–0.9067). The forest plots in Fig. 7 depict the MR analysis results for TIMP4 and SA in the deCODE database. After conducting heterogeneity and pleiotropy tests, as well as MR-PRESSO tests, no significant anomalies were observed (see Supplementary Table S34, S35, and Table S36, respectively). Subsequently, we conducted separate colocalization analyses, and the results showed that in the UKB-PPP database, **TIMP4** (PH4 = 96.1%) had strong evidence of colocalization. In contrast, in the deCODE database, **TIMP4** (PH4 = 97.4%) had strong evidence of colocalization. For details, please refer to Supplementary Table S37.

*Validation group* Firstly, we conducted a global MR scan of the Fenland pQTLs and SA, with the IVs listed in Supplementary Table S47. The final analysis results can be found in Supplementary Table S48. Unfortunately, after FDR correction, no positive results were observed, except for **TIMP4** protein, which had the smallest P-value in the IVW method before correction (*P* = 6.27 × 10–5, P_adjust_ = 0.3353). We considered it to have a potential causal relationship with SA(79) and included it in the subsequent hyprcoloc analysis. In the SMR analysis of eQTLs, two positive genes were identified after FDR correction (**PRIM1**, P_adjust_ = 0.0069, P_HEIDI_ = 0.0977; **BMP8 A**, P_adjust_ = 0.0357, P_HEIDI_ = 0.9352). In the SMR analysis of mQTLs, only one positive gene (**PRIM1**, P_adjust_ = 0.0364, P_HEIDI_ = 0.2652) was identified after FDR correction. Unfortunately, neither the eQTL nor mQTL analysis showed positive results for the protein **TIMP4** after FDR correction, although its P-value was < 0.05 before correction (eQTL, *P* = 0.0455, P_adjust_ = 0.7247; mQTL, *P* = 0.0012, P_adjust_ = 0.4509). For the gene **BMP8 A**, positive results were only observed in the mQTL analysis but not in the FDR-corrected results (*P* = 0.0296, P_adjust_ = 0.6912). We considered the possibility of a potential causal relationship [79] and included these results in the hyprcoloc analysis (mQTL analysis results in Supplementary Table S49, eQTL analysis results in Supplementary Table S50). In the hyprcoloc analysis, we conducted the following analyses (results in Supplementary Table S51): Fenland protein data, mQTL data, and eQTL data of the **TIMP4** protein with SA; mQTL data and eQTL data of the **TIMP4** protein with SA; mQTL data and eQTL data of the **PRIM1** gene with SA; mQTL data and eQTL data of the **BMP8 A** gene with SA. Ultimately, we found strong evidence of colocalization between the **TIMP4** protein in Fenland pQTLs and SA (P_posterior_ = 0.8492); no colocalization was found between the protein and SA in the mQTL and eQTL data. For the **PRIM1** gene, we found solid evidence of colocalization with SA in both mQTL and eQTL data (P_posterior_ = 0.9981). The **BMP8 A** gene showed strong evidence of colocalization with SA only in the eQTL data (P_posterio_r = 0.9266). Results of the heterogeneity and pleiotropy tests in the global MR scan of Fenland protein pQTLs and SA can be found in Supplementary Table S52, and the MR-PRESSO test results are presented in Supplementary Table S53.

*Supplementary analysis* In our bidirectional MR study on rQTL and SA, we found that after FDR correction, no positive results were observed in the forward analysis (with SA as the outcome) (overall results in Supplementary Table S54). However, in the reverse analysis (with SA as the exposure) (overall results in Supplementary Table S55), we identified 24 protein ratios with a causal relationship with SA, of which 11 showed positive results primarily with the IVW method (refer to Table 2). Heterogeneity and pleiotropy in the forward MR analysis are detailed in Supplementary Table S56 and Supplementary Table S57. In contrast, those in the reverse MR analysis are presented in Supplementary Table S58 and Supplementary Table S59.

### PheWAS analysis

*Search for phenotypic associations based on gene enrichment* After a series of trait screenings in the UK Biobank dataset, we identified 34 statistically significant associations with SA after FDR correction (details can be found in Supplementary Table S7). Among them, two types of traits caught our interest: impedance of the body (impedance of leg (right), P_adjust_ = 0.0113; impedance of whole body, P_adjust_ = 0.0126) and predicted body mass (arm predicted mass (right), P_adjust=_0.0014; arm predicted mass (left), P_adjust_ = 0.0126; leg predicted mass (left), P_adjust_ = 0.0187; trunk predicted mass, P_adjust_ = 0.0113).

**Fig. 8:**
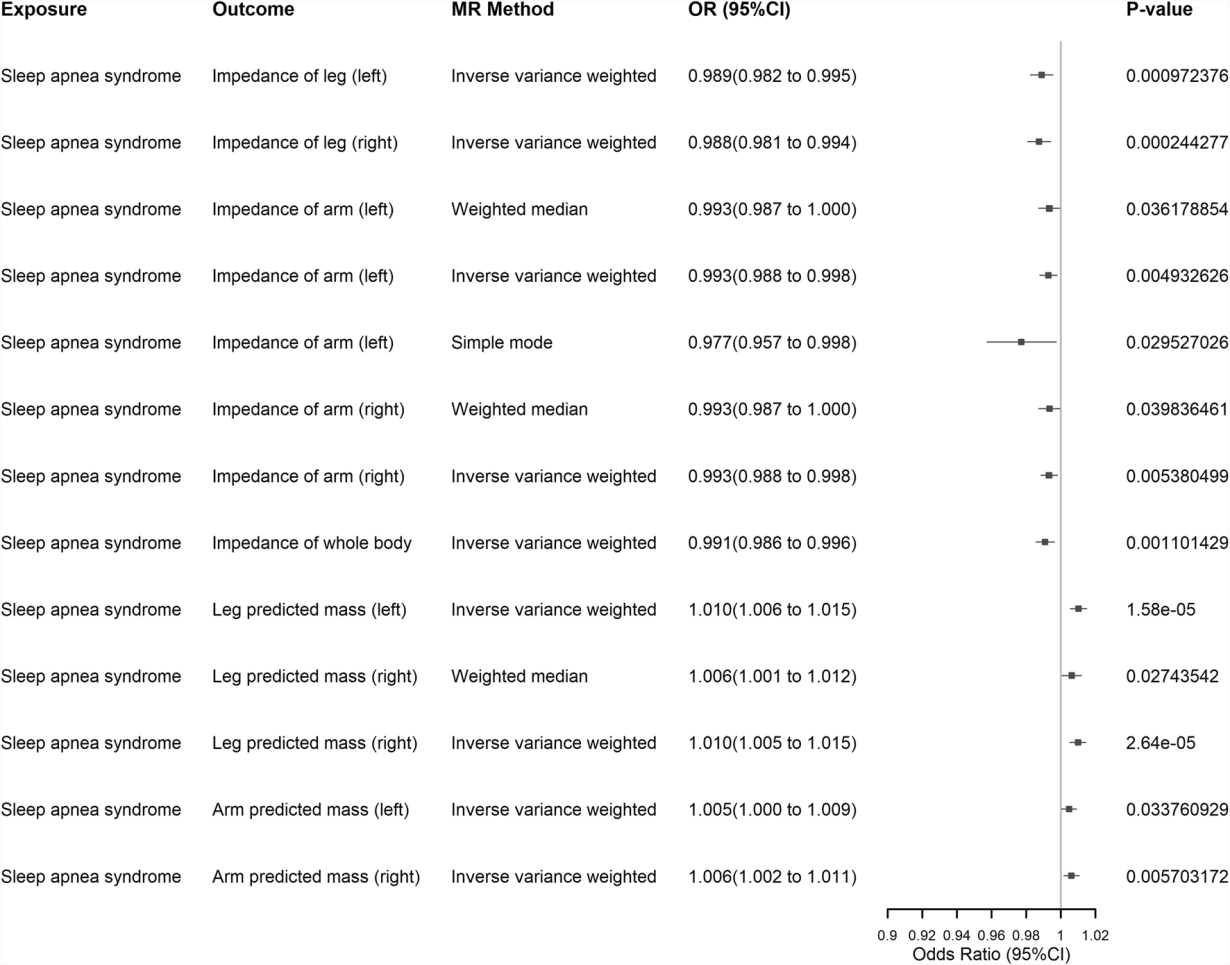
Forest map of positive MR Analysis of 10 factors and SA (including at least one MR Analysis method of IVW method). Exposure: Signifies our exposure factor as sleep apnea. Outcome: Indicates our outcome factors as body impedance factors and body predicted mass factors. MR Method: Denotes the analytical method employed in our study. OR (95% CI): Represents the odds ratio and its corresponding 95% confidence interval. P-value: Signifies the p-value obtained from the respective method of analysis. SA: Sleep apnea; MR: Mendelian Randomization; IVW: Inverse Variance Weighted

**Fig. 9:**
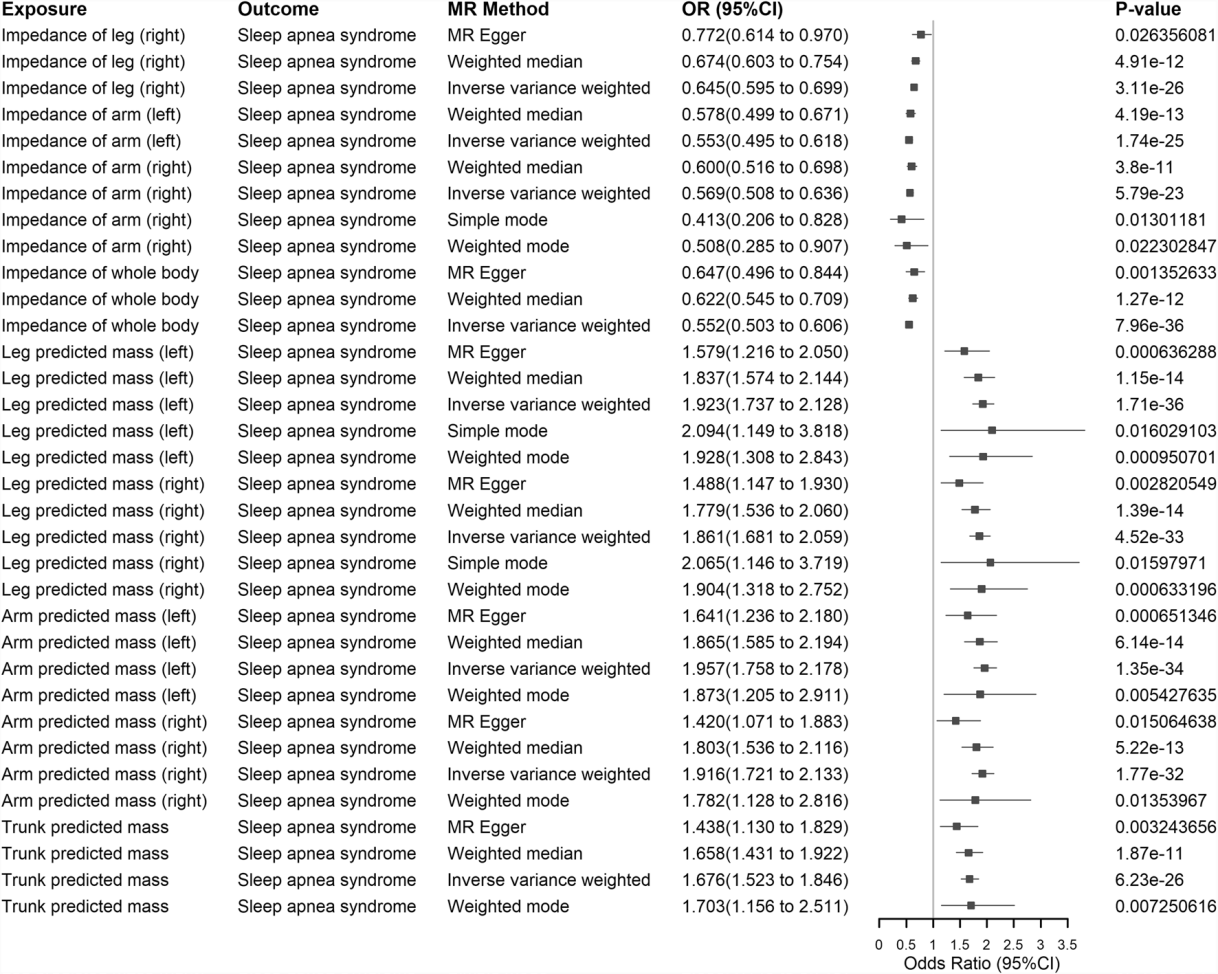
Results of reverse MR Analysis of 10 factors and SA (at least one MR Analysis method including IVW method). Exposure: Signifies our exposure factor as body impedance factors and body predicted mass factors. Outcome: Indicates our outcome factors as sleep apnea. MR Method: Denotes the analytical method employed in our study. OR (95% CI): Represents the odds ratio and its corresponding 95% confidence interval. P-value: Signifies the p-value obtained from the respective method of analysis. SA: Sleep apnea; MR: Mendelian Randomization; IVW: Inverse Variance Weighted

*MR and LDSC Analysis* Considering that a total of 10 factors, including impedance of the body (impedance of arm (left), impedance of arm (right), impedance of leg (left), impedance of leg (right) and impedance of whole body) and predicted body mass (arm predicted mass (left), arm predicted mass (right), leg predicted mass (left), leg predicted mass (right) and trunk predicted mass) may potentially have causal relationships with SA, we performed bidirectional MR analysis. We selected the results of at least one MR method, including the IVW method. In the forward MR analysis (with SA as the exposure factor), we found that all factors except for trunk predicted mass showed a causal relationship with SA (see Supplementary Table S38 and Fig. 8). In the reverse MR analysis (with SA as the outcome factor), all factors except for the impedance of the leg (right) exhibited a causal effect on SA (see Supplementary Table S39 and Fig. 9). We also conducted heterogeneity and pleiotropy tests, as well as MR-PRESSO analysis (heterogeneity, pleiotropy, and MR-PRESSO analysis for forward MR analysis are shown in Supplementary Table S40, S41, and S42, respectively; heterogeneity, pleiotropy, and MR-PRESSO analysis for reverse MR analysis are shown in Supplementary Table S43, S44, and S45, respectively). We found that both the forward and reverse MR analyses showed heterogeneity, and therefore, we employed the IVW method using a random-effects model [80, 81], which still yielded significant results. Additionally, most of these 10 factors exhibited pleiotropy in the forward MR analysis, and even after removing outliers, pleiotropy was still present. However, in the reverse MR analysis, the results were the opposite, with most factors showing no pleiotropy. After FDR correction, the MR analysis results remained statistically significant. To further validate the robustness of our results, we performed LDSC analysis which revealed significant genetic correlations between SA and all 10 factors, namely: SA and impedance of leg (right) (r_g_ = − 0.4001, *P* = 1.40 × 10^−22^), SA and impedance of leg (left) (r_g_ = − 0.4048, *P* = 3.79 × 10^−22^), SA and impedance of arm (right) (r_g_ = − 0.4181, *P* = 1.43 × 10^−22^), SA and impedance of arm (left) (r_g_ = − 0.4202, P = 2.68 × 10^−22^), SA and impedance of whole body (r_g_ = − 0.4408, *P* = 3.35 × 10^−26^), SA and leg predicted mass (right) (r_g_ = 0.3765, *P* = 1.44 × 10^−21^), SA and leg predicted mass (left) (r_g_ = 0.3967, *P* = 8.28 × 10^−24^), SA and arm predicted mass (right) (r_g_ = 0.3615, *P* = 5.27 × 10^−22^), SA and arm predicted mass (left) (r_g_ = 0.3813, *P* = 3.95 × 10^−23^) and SA and trunk predicted mass (r_g_ = 0.2746, *P* = 1.61 × 10^−13^). Detailed data can be found in Supplementary Table S46.

## Discussion

This study allows us to gain a deeper understanding of the relationship between genetic variations, phenotypes, and gut microbiota, thereby expanding our understanding of SA pathogenesis. Additionally, it helps identify potential drug targets and biomarkers for SA, including causal relationships and dist interactions under a genomic background. Our investigation on gut microbiota and inflammatory proteins revealed that microbial taxa at the 24 genus level and 13 inflammatory proteins are causally related to SA. In the pQTL analysis, we discovered **TIMP4** as a novel protein target. Furthermore, through validation in the mQTL and eQTL validation groups, we identified **PRIM1** and **BMP8 A** as potentially influencing SA. Additionally, PheWAS analysis shows associations between SA and body impedance traits and predicted body mass. These findings offer valuable insights for intervention strategies and the development of potential therapeutic targets for SA.

Recent studies by Burgess et al. [82] have highlighted five common pitfalls in MR investigations: (1) inappropriate research questions, (2) improper selection of genetic variants as instruments, (3) insufficient interrogation of results, (4) inappropriate interpretation of results, and (5) lack of engagement with prior work. Our study has carefully addressed these pitfalls. First, we implemented rigorous controls in the selection of IVs, including a detailed rationale for adjusting the p-value threshold from 5 × 10^−8^ to 5 × 10^−5^ and validating our IVs using the NCBI database to ensure strong relevance. While our primary focus is on SA, the outcome variables in our PheWAS analysis are human physiological metrics, and the exposures in our QTL analyses are genes, both of which align well with biological mechanisms. Although the gut microbiome may not always be an ideal exposure for MR studies, we mitigated this limitation by linking our findings to the TIMP4 gene pathway in the discussion. Thus, while some aspects of our analysis may partially fall into the trap of inappropriate exposure, our overall study design avoids the pitfall of inappropriate research questions. Second, Burgess et al. emphasized selecting genetic variants based on their biological relevance to the risk factors of interest [82]. In our QTL analyses, this issue is inherently addressed because the exposures are genes themselves, which have well-defined biological relationships. Similarly, in our PheWAS analysis, the exposures are single or a few SNPs, selected based on stringent criteria and supported by existing literature, ensuring strong biological relevance. Therefore, we have effectively avoided the second pitfall of “improper selection of variants as instruments.” Third, to address potential concerns of “insufficient interrogation of results” we employed robust methods, including sensitivity analyses, colocalization analyses, HEIDI tests, and hyprcoloc analyses, to enhance the credibility of our conclusions. These approaches align with the recommendations of Burgess et al. [82] Fourth, we provided comprehensive interpretations of our results. In multi-omics studies, where exposures are genes and outcomes are diseases, our interpretations are supported by extensive literature on gene-disease pathways. For the gut microbiome, due to its complex associations, we linked our findings to pathways and integrated them with our omics results, particularly focusing on TIMP4. However, for PheWAS results, which involve human physiological metrics, we acknowledged that simple causal descriptions may not suffice and instead provided cautious interpretations and outlined future research directions. Finally, while our PheWAS results currently lack extensive literature support, we explicitly addressed this limitation by emphasizing the need for rigorous randomized controlled trials and basic research to validate our findings. In summary, our study is structured into three complementary parts, each designed to address and mitigate the five pitfalls of MR investigations outlined by Burgess et al. This approach enhances the robustness and reliability of our conclusions.

The study suggests that dysbiosis of the gut microbiota may contribute to oxidative stress, upregulation of hypoxia-inducible factor-1α and downregulation of epithelial tight junction protein expression, leading to the occurrence of intestinal hypoxia (IH) in the body [83]. This induces a cycle of hypoxia/reoxygenation, under which an increased duration of hypoxia favors the growth of obligate anaerobic bacteria, consequently altering the composition of the microbiota [84]. The coexistence of OSA and systemic hypoxia perpetuates a vicious cycle. The hypoxic state inherent to OSA can lead to gut dysbiosis, which in turn exacerbates intestinal hypoxia and potentially systemic hypoxia, further aggravating the OSA condition. On the other hand, some evidence suggests that oxygenation levels can alter the composition of the gut microbiota and its interaction with the host by modulating intestinal epithelial permeability [85–88]. This can lead to the translocation of microbial-derived molecules into the bloodstream.

Additionally, recurrent arterial hypoxemia events caused by upper airway obstruction/collapse, which is one of the mechanisms underlying OSA pathogenesis, may have a negative impact on gut function and induce significant changes in the gut microbiome [89]. These changes include an increase in Prevotella, Veillonella, Desulfovibrio, and Lachnospiraceae, as well as a decrease in Bacteroidetes, Firmicutes, Turicibacter, Ruminococcaceae, and Enterococcaceae [90]. The genus Holdemania, which we investigated in our study, belongs to the family Lachnospiraceae and is a Gram-positive bacteria. It is a less-studied bacterium, and thus, there needs to be more knowledge about its biological characteristics and ecological functions. Furthermore, some studies have found that the death products of certain gram-negative gut bacteria (endogenous lipopolysaccharide(LPS)) can translocate into the capillaries through a TLR4-dependent mechanism, potentially contributing to the development of SA [91]. Therefore, it is evident that there is a bidirectional relationship between the gut microbiota and SA, which aligns with the findings of our bidirectional MR analysis.

In our study, the C–C motif chemokine 28 belongs to the chemokine family, which regulates the expression and structure of target cell adhesion molecules and cytoskeletal proteins to guide various cells, especially leukocytes, to migrate along concentration gradients [92, 93]. Chemokine-directed leukocyte migration can also contribute to diseases with immune or inflammatory components [92, 93]. As mentioned earlier, the inflammatory response may induce intestinal hypoxia (IH) in the body, ultimately leading to the development of OSA. Research suggests that a prothrombotic state may impact the coagulation status of patients with OSA, potentially fostering the progression of atherosclerosis [94] and thereby potentially contributing to the development of adverse cardiovascular complications associated with OSA. These coagulation alterations appear to involve the plasminogen activation system, with urokinase-type plasminogen activator, a component of this system [95], identified in our mediation analysis. In our study, there is a mediating effect between the genus Holdemania and Urokinase-type plasminogen activator levels in relation to SA. Although existing observational studies have not yet established a causal relationship, previous research has suggested the involvement of genus Holdemania in impaired lipid metabolism [96], which may increase the risk of thrombosis [97, 98]. As mentioned earlier, Urokinase-type plasminogen activator levels are involved in the fibrinolysis process. Taken together, one possible mechanism is that genus Holdemania, through impaired lipid metabolism, increases the risk of thrombosis, ultimately affecting Urokinase-type plasminogen activator levels and potentially influencing the occurrence of OSA. However, the specific direct mechanisms between urokinase-type plasminogen activator and SA require further in-depth investigation for elucidation.

Currently, four **TIMPs** have been identified in vertebrates (**TIMP1** to **TIMP4**) [99]. One significant role of **TIMPs** in tissue remodeling is their ability to inhibit matrix proteases by forming 1:1 enzyme-inhibitor complexes. The expression of **TIMPs** in tissue remodeling and under physiological conditions is also tightly regulated to maintain the balance of extracellular matrix metabolism [100, 101]. Disruption of this balance may lead to diseases associated with uncontrolled matrix turnover. Obesity has been well-established as a risk factor for OSA [102]. In a study investigating nutrient-induced obesity in mice, it was observed that the expression of **TIMP4** was downregulated in obese mice [103]. Regarding **TIMP4**, research suggests that it can modulate the activity of A Disintegrin and Metalloproteinase (**ADAM**) 10, thereby affecting platelet thrombus formation [104]. This may involve the previously mentioned Urokinase-type plasminogen activator levels, ultimately impacting the risk of OSA. In the bioinformatics analysis, we identified an interesting signaling pathway, the Notch signaling pathway, which involves **DLL4** stimulation of the renal cell line caki-1, leading to overexpression of **MMP2** and **MMP9**, metalloproteinases closely associated with the metastatic process [105]. This process may involve changes in the activity of **TIMP4** and the **TIMP** family. Additionally, the initial cleavage of the ligand protein in this pathway is catalyzed by the ADAM family of metalloproteinases, implicating the regulatory role of the **TIMP** family on **ADAM** [105]. Furthermore, this pathway can trigger changes in the renin-angiotensin system (RAS) through renin alteration [106], leading to changes in inflammatory factors, potentially causing oxidative stress and hypoxia, which can affect the critical mechanism IH in OSA. In conclusion, **TIMP4** may influence the risk of OSA occurrence through the complex mechanisms of the Notch signaling pathway. Unfortunately, there is currently no evidence to establish the direct relationship between **TIMP4** and SA. Our study may serve as a foundational step towards unveiling this association, and it is hoped that in the future, research should aim to investigate the association between **TIMP4** and SA further. **PRIM1** (DNA Primase Subunit 1) is a protein-coding gene that encodes a component of the DNA polymerase α complex [107]. Our previous mQTL and eQTL range MR analysis of SA found strong support for the colocalization of SA with **PRIM1**. Research has indicated that **PRIM1** can induce ubiquitination and degradation of p53 by upregulating ubiquitin-conjugating enzyme E2 C (**UBE2 C**). P53, in turn, supports cellular function by coordinating anti-inflammatory responses. The downregulation of p53 may lead to inflammation, thereby increasing the risk of OSA. Furthermore, we found that the p53 signaling pathway can activate neuroinflammatory responses and impair mitochondrial function through the generation of reactive oxygen species (ROS), thereby inducing cellular apoptosis [108]. The neurocognitive impairments associated with SA are primarily triggered by chronic IH (CIH)-induced neuroinflammation and oxidative stress [109]. Notably, mitochondrial ROS (mtROS) plays a critical role in hypoxia-related tissue damage [109]. Thus, the p53 signaling pathway, mediated by **PRIM1**, may increase the risk of SA not only through inflammatory mechanisms but also indirectly by accelerating mitochondrial dysfunction. However, the mechanism by which **PRIM1** influences gene expression and subsequently affects SA risk through the methylation of cg23183521 remains unclear. Further observational studies and experimental validation are necessary to elucidate this relationship. **BMP8 A** (Bone Morphogenetic Protein 8a) is a protein-coding gene that encodes a secreted ligand of the TGF-β (Transforming Growth Factor-beta) superfamily. In our eQTL SMR analysis, we found strong support for the colocalization of SA with **BMP8 A**. In the previous bioinformatics analysis, enrichment analysis of the **BMP8 A** gene pathways and processes indicated significant enrichment of these genes in the positive regulation of pathway-restricted SMAD protein phosphorylation biological process [110]. Existing evidence suggests that the upregulation of SMAD protein phosphorylation pathways is positively correlated with the regulation of the oncogenic TGF-β signaling pathway [111]. This association may be attributed to increased **BMP8 A** expression leading to elevated levels of TGF-β, thereby exacerbating inflammatory responses in OSA patients.

A study conducted at Stanford University School of Medicine found that leg bioimpedance can predict an individual’s risk of developing heart failure in the future [112]. Due to the higher prevalence of obesity among patients with SA [102], particularly those with OSA, this typically correlates with increased impedance values. Additionally, airway obstruction in sleep apnea results in a loss of normal muscle tone [113], which may suggest a positive correlation between body impedance and sleep apnea. However, our study found a negative correlation between body impedance and SA. Possible explanations for this discrepancy include fluid loss leading to reduced body water content or increased muscle tension, necessitating further clinical trials for validation. On the other hand, another significant factor in our study, body-predicted mass, showed a positive correlation with SA. One possible explanation for this is that a considerable number of SA patients are obese [2, 102], resulting in higher body-predicted mass values. However, there is currently limited research in this area, and further exploration is needed to elucidate whether other factors contribute to this relationship. Additionally, the potential of body-predicted mass as a predictive factor for adverse events in SA occurrence requires further investigation.

However, this study also has certain limitations. Firstly, the gut microbiome and inflammatory proteins can be influenced by demographic factors, diet, or medication. The presence of heterogeneity and reduced inter-individual variability diminishes the statistical power and robustness of the results. Additionally, the majority of individuals in the GWAS studies are of European ancestry, with limited participation from other racial groups, which may introduce slight biases and affect the generalizability of the findings. Furthermore, the number of SNPs available for genome-wide analysis is limited. To address this issue, we employed a slightly relaxed threshold (*p* < 5 × 10^−5^) for our MR analysis, which is commonly used in other studies. However, since the selected IVs did not reach the conventional GWAS significance threshold (*p* < 5 × 10^−8^), there is a possibility of increased false positives. All selected SNPs had an f-statistic exceeding 10, indicating the robustness of our IVs. However, these measures do not completely overcome the limitations in terms of SNP quantity. A fourth limitation is that the precision of MR estimates partly depends on the sample size, and as such, expanding the sample size and data sources is necessary for SA. A fifth limitation is the potential unavailability of specific pQTL associations for certain proteins, considering the limited accessibility of pQTL data. Although we concurrently analyzed all proteins in the UKB-PPP and deCODE databases, using Fenland cohort protein data for validation, supplemented by non-proteomic approaches (mQTL and eQTL) for reinforcement, the analysis of **TIMP4** protein in the Fenland cohort with SA yielded negative results after FDR correction, despite its strong colocalization with SA. Similarly, the non-proteomic data also yielded negative adjusted results, which did not support our validation findings, further restricting access to data for all other proteins potentially associated with SA. In addition, our PheWAS study only involved data from the UK Biobank database, and we cannot exclude the possibility that other databases may have additional predictive factors for SA. Furthermore, in the MR analysis following gene enrichment, our results exhibited noticeable heterogeneity and pleiotropy, which undermines the robustness of our findings. Therefore, we used LDSC analysis to strengthen the genetic correlation between SA and body impedance and body predicted mass, which provides relatively robust results. We also have a limitation in that our study is divided into three parts: gut microbiome, proteome, and PheWAS. However, we are unable to organically integrate these parts in light of our findings. This encourages us to conduct further in-depth research to identify factors that can balance or correlate in order to integrate our findings. Moreover, while MR analysis provides valuable etiological insights, it is essential to validate our findings through rigorous RCT and basic research before applying them in clinical practice.

## Conclusion

In summary, our bidirectional MR analysis identified 24 gut microbial taxa and 13 inflammatory proteins causally linked to SA, with C–C motif chemokine 28 showing significant bidirectional causality. We uncovered a mediating pathway, “Holdemanella genus-urokinase-type plasminogen activator levels-SA,” and highlighted **TIMP4** as a potential therapeutic target through pQTL analysis. Validation groups implicated **PRIM1** and **BMP8 A** as genes influencing SA. PheWAS identified body impedance and predicted body mass as significant predictors of adverse SA events, encompassing 10 specific factors. Future work will focus on experimental validation of proteins/genes like **TIMP4**, exploring interactions between gut microbiota, inflammatory proteins, and identified genes, and integrating findings into clinical trials to assess real-world applications, such as TIMP4’s therapeutic potential. The path forward remains extensive and promising.

## Data availability statement

All original data sources referenced in this study are appropriately cited within the manuscript. Representative analysis code examples have been archived in Supplementary Material 2, comprising implementations for: (1) gut microbiome-disease associations; (2) inflammatory protein-disease relationships; (3) gut microbiome-inflammatory protein interactions; (4) Mendelian Randomization (MR) analysis of Fenland protein-disease associations; (5) hyprcoloc analytical workflow; and (6) colocalization analysis framework. Code implementation details are omitted for the following cases: (1) SMR analysis: Full methodological specifications are provided in the Methods section. The SMR analysis method is described in the Methods section; the SMR software (SMR-1.3.1) is available from the Yang Lab (https://yanglab.westlake.edu.cn/software/smr/#ExecutableFiles(version1.3.1)), including parameter selection and usage instructions (https://yanglab.westlake.edu.cn/software/smr/#SMR&HEIDIanalysis). (2) PheWAS analysis: Conducted using the R package ‘mrasst’ (v0.2.0), with comprehensive results from SMR analyses and GWAS/QTL associations for prioritized SNPs presented in Supplementary Tables. Raw datasets were derived from third-party repositories as specified in the manuscript. Readers are directed to the original sources for data access requests. Researchers may contact the corresponding author for clarifications regarding code implementation.

## Supplementary Information

Supplementary file 1

Supplementary file 2

Supplementary file 3

Supplementary file 4

## Abbreviations

ADAM: A disintegrin and metalloproteinase
BMP8 A: Bone morphogenetic protein 8a
BMI: Body mass index
CIH: Chronic intermittent hypoxia
EAF: Effect allele frequency
eQTL: Expression quantitative trait loci
FDR: False discovery rate
GWAS: Genome-wide association study
HEIDI test: Heterogeneity in dependent instruments test
Hyprcoloc: Hypothesis prioritization analysis for multi-trait colocalization
IVs: Instrumental variables
IVW: Inverse variance weighted
LD: Linkage disequilibrium
LDSC: Linkage disequilibrium score regression
LPS: Lipopolysaccharide
MAF: Minor allele frequency
MMPs: Matrix metalloproteinases
MQTL: Methylation quantitative trait loci
MR: Mendelian randomization
mtROS: Mitochondrial reactive oxygen species
NCBI: National center for biotechnology information
OSA: Obstructive sleep apnea
PheWAS: Phenome-wide association study
PH4: Statistical probability of hypothesis H4
PRIM1: DNA primase subunit 1
PRS: Polygenic risk scores
PQTL: Protein quantitative trait loci
RAS: Renin-angiotensin system
RFPRS: Risk factor polygenic risk scores
RCT: Randomized controlled trial
ROS: Reactive oxygen species
SA: Sleep apnea
SMR: Summary data-based mendelian randomization
SNPs: Single nucleotide polymorphisms
TGF-β: Transforming growth factor-beta
TIMPs: Tissue inhibitors of metalloproteinase
TSVD: Truncated singular value decomposition
UBE2 C: Ubiquitin-conjugating enzyme E2 C
UKB-PPP: UK biobank pharma proteomics project
WM: Weighted median
