# Supplementary figures and images for: Integrative multi-omics investigation of sleep apnea: gut microbiome metabolomics, proteomics and phenome-wide association study
Source: Nutr Metab (Lond). 2025 Jun 10;22:57. doi: 10.1186/s12986-025-00925-0 (PMC12150496; doi:10.1186/s12986-025-00925-0)

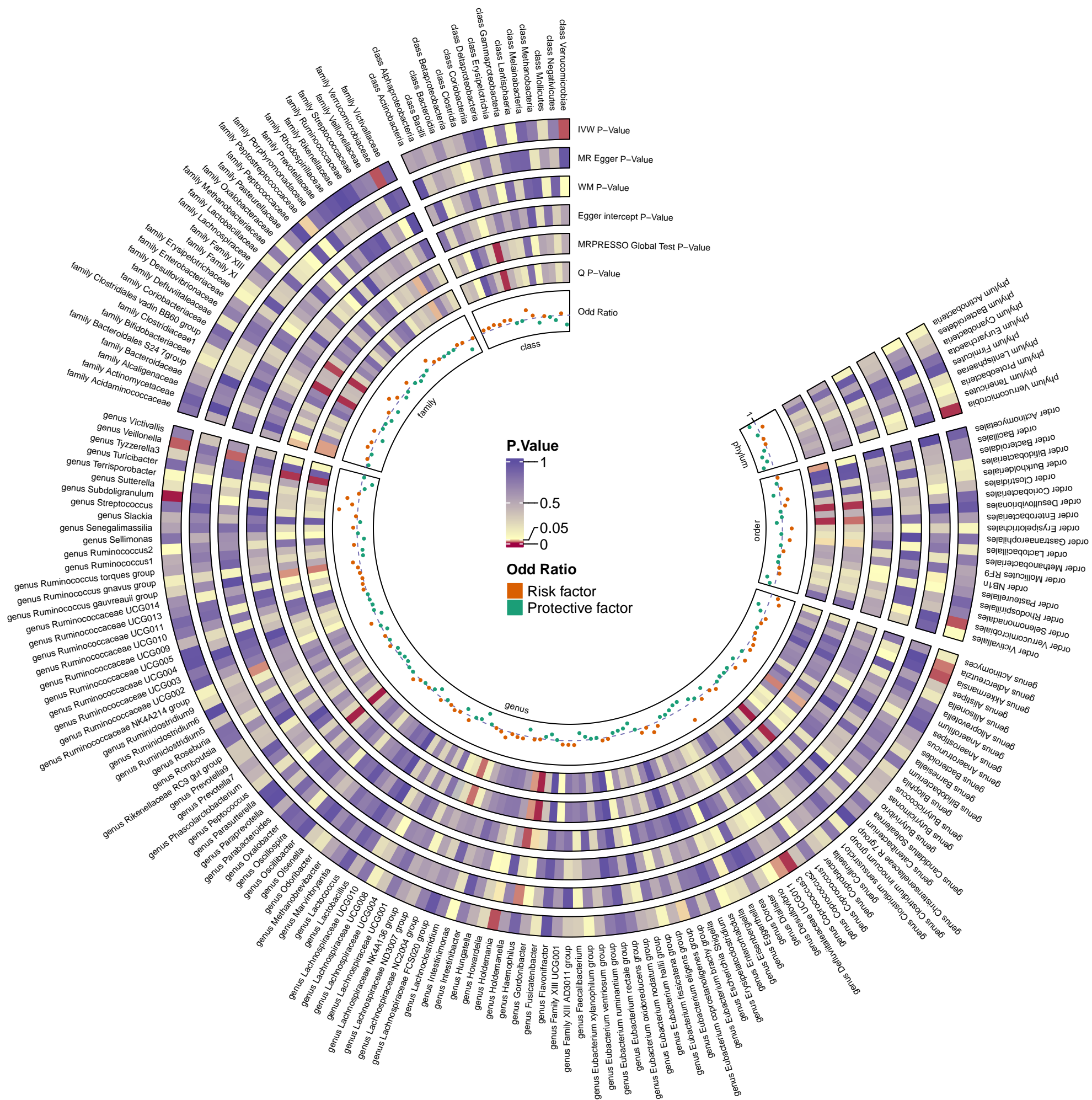

Supplement: Supplementary file 1 [file 12986_2025_925_MOESM1_ESM.pdf]
