# Supplementary material for: Integrative multi-omics investigation of sleep apnea: gut microbiome metabolomics, proteomics and phenome-wide association study
Source: Nutr Metab (Lond). 2025 Jun 10;22:57. doi: 10.1186/s12986-025-00925-0 (PMC12150496; doi:10.1186/s12986-025-00925-0)
Supplement: Supplementary file 2 [file 12986_2025_925_MOESM2_ESM.docx]

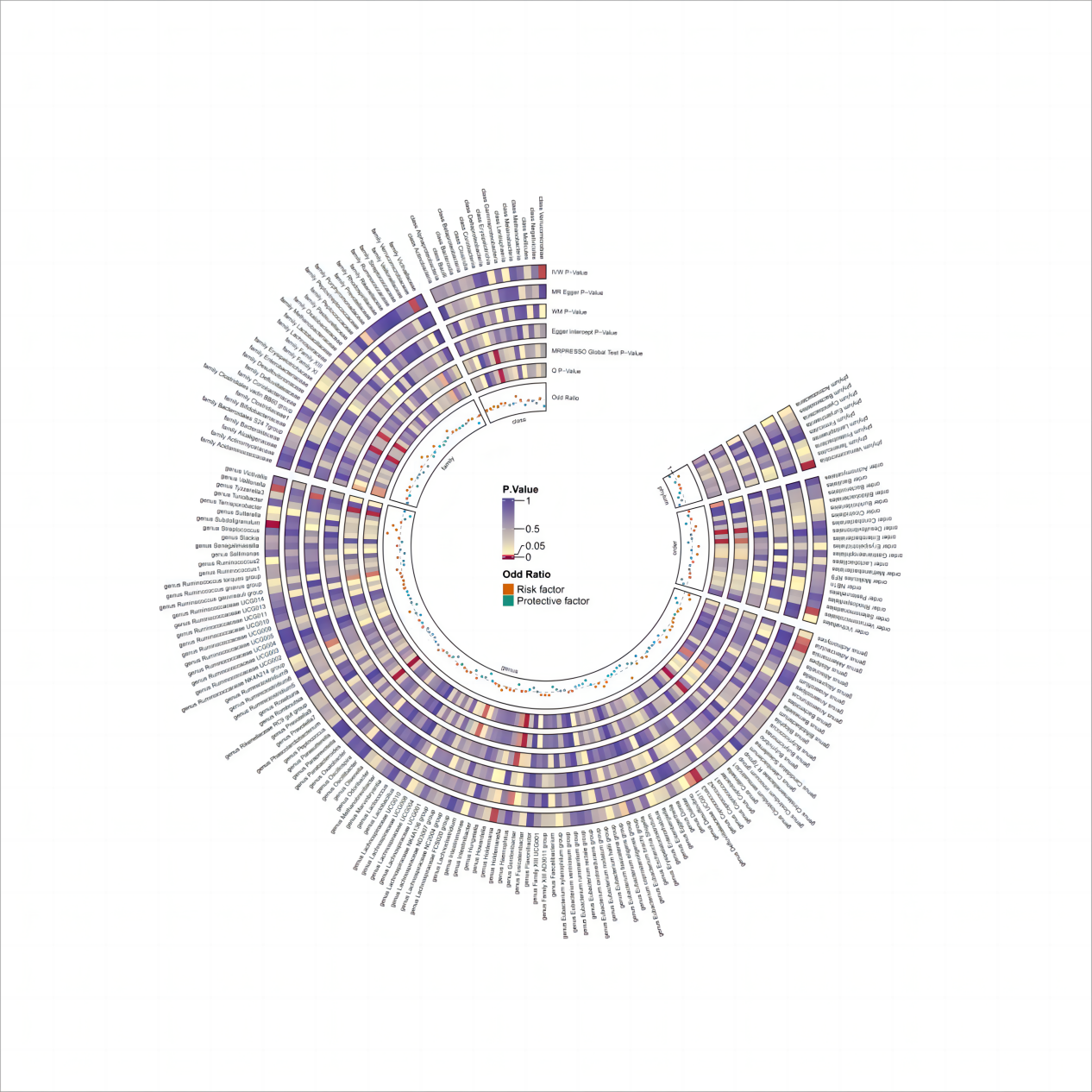


**Supplementary Figure 1: Circular heatmap representing the results of the MR analysis of gut microbiota and SA.**


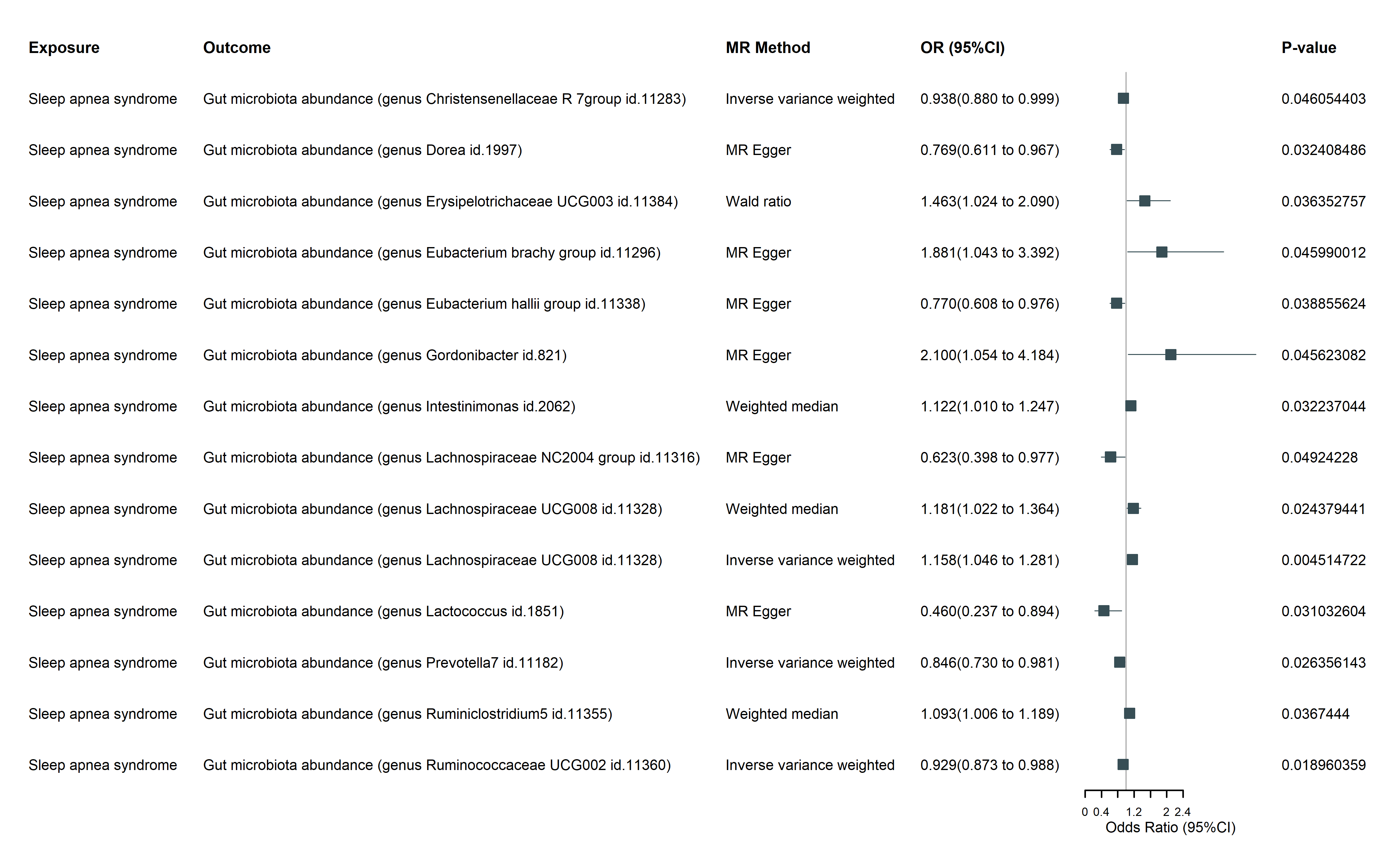
**Supplementary Figure 2: Forest maps of 13 genus-level gut microbes in reverse MR Analysis with significant causal relationship to SA . Exposure: This signifies our exposure factor as sleep apnea. Outcome: Indicates our outcome factors as gut microbiota. MR Method: Denotes the analytical method employed in our study. OR (95% CI): Represents the odds ratio and its corresponding 95% confidence interval. P-value: Signifies the p-value obtained from the respective method of analysis.**

**
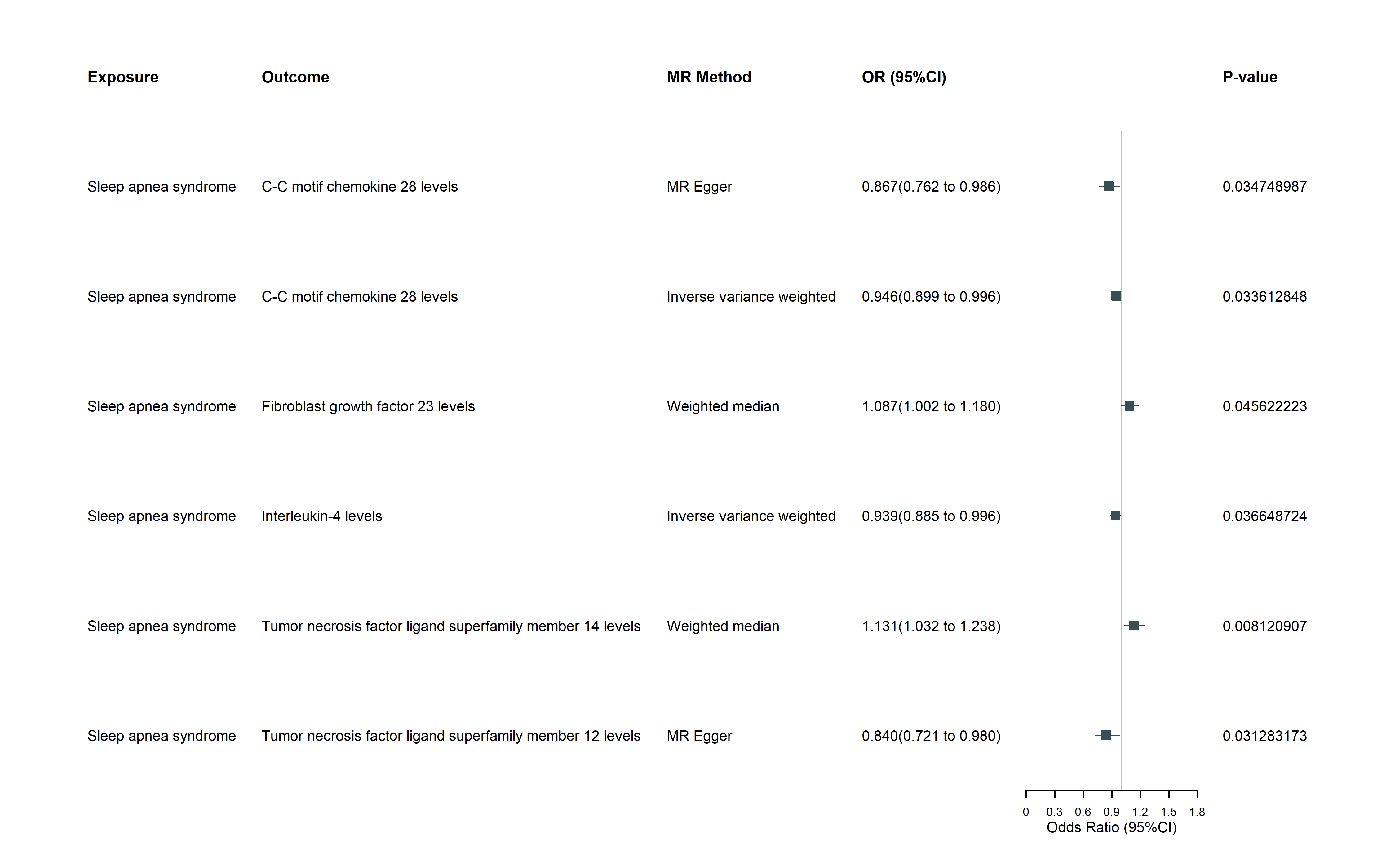
Supplementary Figure 3: Forest map of 5 inflammatory proteins in reverse MR Analysis with significant causal relationship with SA. Exposure: This signifies our exposure factor as sleep apnea. Outcome: Indicates our outcome factors as inflammatory proteins.. MR Method: Denotes the analytical method employed in our study. OR (95% CI): Represents the odds ratio and its corresponding 95% confidence interval. P-value: Signifies the p-value obtained from the respective method of analysis.**
