# Supplementary material for: Integrative multi-omics investigation of sleep apnea: gut microbiome metabolomics, proteomics and phenome-wide association study
Source: Nutr Metab (Lond). 2025 Jun 10;22:57. doi: 10.1186/s12986-025-00925-0 (PMC12150496; doi:10.1186/s12986-025-00925-0)
Supplement: Supplementary file 3 [file 12986_2025_925_MOESM3_ESM.docx]

**The code content includes:**

1. **Gut microbiome to disease.**
2. **Inflammatory proteins to disease.**
3. **Gut microbiome to inflammatory proteins.**
4. **MR Analysis of Fenland protein for disease.**
5. **Example code for hyprcoloc analysis.**
6. **Example code for colocalization analysis.**

#######**1. Gut microbiome to disease**

library(stringr)

library(data.table)

library(TwoSampleMR)

library(MRPRESSO)

library(dplyr)

library(ggsci)

library(ggplot2)

library(TSMRhelper)

library(forestploter)

library(grid)

library(stringr)

library(grDevices)

# outcome

#1.Online outcome

id<-"ebi-a-GCST90018916"

#2.Local outcome

id<-""

outcome_all<-fread("x.gz",data.table = F)

# Obtain exposure to intestinal flora

ao<-read.csv("ao.csv")

id_exp<-subset(ao,pmid==33462485)

id_exp<-id_exp[!str_detect(id_exp$trait,"unknown"),]

exp<-list()

i <- 1

for (ids in id_exp[1:nrow(id_exp),]$id) {

print(ids)

while(TRUE){

message_to_next <<- TRUE

error_to_next <<- FALSE

try({withCallingHandlers(exp_clumped<-extract_instruments(outcomes = ids,

p1 = 1e-05,

clump = TRUE,

r2 = 0.001,

kb = 10000,

access_token = NULL),

message = function(c) if (stringr::str_detect(as.character(c),"Failed to")) message_to_next <<- FALSE)

error_to_next <<- TRUE})

if(message_to_next == TRUE&error_to_next == TRUE) { break }

}

if(is.null(exp_clumped))next

exp_clumped$exposure<-id_exp[id_exp$id==exp_clumped$id.exposure[1],]$trait

exp[[i]]<-exp_clumped

i<-i+1

}

exp_clumped<-do.call(rbind,exp)

# Preservation exposure

write.csv(exp_clumped,file = "exp_clumped.csv",row.names = F)

# Get outcome

if(id==""){

snp<-unique(exp_clumped$SNP)

outcome<-outcome_all[outcome_all$rsids%in%snp,]

outcome_dat<-format_data(outcome,

type = "outcome",

beta_col = "beta",

se_col = "sebeta",

effect_allele_col = "alt",

other_allele_col = "ref",

snp_col = "rsids",

pval_col = "pval")

}else{

while(TRUE){

message_to_next <<- TRUE

error_to_next <<- FALSE

try({withCallingHandlers(outcome_dat <- extract_outcome_data(snps = exp_clumped$SNP,

outcomes = id,

proxies = F,

access_token = NULL

),

message = function(c) if (stringr::str_detect(as.character(c),"Failed to")) message_to_next <<- FALSE)

error_to_next <<- TRUE})

if(message_to_next == TRUE&error_to_next == TRUE) { break }

}

}

write.csv(outcome_dat,file = "outcome.csv",row.names = F)

dat<-harmonise_data(exposure_dat = exp_clumped,outcome_dat = outcome_dat)

dat<-dat[dat$mr_keep,]

write.csv(dat,file = "dat.csv",row.names = F)

# mr

res<-generate_odds_ratios(mr(dat))

write.csv(res,file = "res.csv",row.names = F)

# Heterogeneity analysi

heterogeneity<-mr_heterogeneity(dat)

write.csv(heterogeneity,file = "gutto91_heterogeneity.csv",row.names = F)

#pleiotropy_test

pleiotropy_test<-mr_pleiotropy_test(dat)

write.csv(pleiotropy_test,file = "gutto91_pleiotropy_test.csv",row.names = F)

# MRPRESSO

mp<-list()

mps<-unique(dat$id.exposure)

i=1

for (mpi in mps) {

data<-dat[dat$id.exposure==mpi,]

if(nrow(data)<4){next}

# MRPRESSO

mp[[i]]<-mr_presso(BetaOutcome = "beta.outcome",

BetaExposure = "beta.exposure",

SdOutcome = "se.outcome",

SdExposure = "se.exposure",

OUTLIERtest = TRUE,

DISTORTIONtest = TRUE,

data = data,

NbDistribution = 1000,

SignifThreshold = 0.05)

mp[[i]]$phe <- data$exposure[1]

i=i+1

}

result_mp_all<- data.frame()

for (i in 1:length(mp)) {

print(mp[[i]]$phe)

result_mp <- mp[[i]]$`Main MR results`

result_mp$RSSobs <- mp[[i]]$`MR-PRESSO results`$`Global Test`$RSSobs

result_mp$Pvalue <- mp[[i]]$`MR-PRESSO results`$`Global Test`$Pvalue

result_mp$phe <- mp[[i]]$phe

result_mp_all <- rbind(result_mp_all,result_mp)

}

table(is.na(result_mp_all$`Causal Estimate`))

write.csv(result_mp_all,file = "gutto91_MRPRESSO.csv")

####### Reverse mr######

outcome_all<-fread("x.csv",data.table = F)

outcome_all = outcome_all[,-1]

colnames(outcome_all) = c("rsids","alt","ref","or","pval","beta","sebeta")

if(id==""){

exp<-outcome_all

exp$pval<-as.numeric(exp$pval)

exp<-subset(exp,pval<1e-05)

exp<-format_data(exp,

snp_col = "rsids",

effect_allele_col = "alt",

eaf_col = "af_alt",

pval_col = "pval",

beta_col = "beta",

se_col = "sebeta")

while(TRUE){

message_to_next <<- TRUE

error_to_next <<- FALSE

try({withCallingHandlers(exp_clumped<-clump_data(exp),

message = function(c) if (stringr::str_detect(as.character(c),"Failed to")) message_to_next <<- FALSE)

error_to_next <<- TRUE})

if(message_to_next == TRUE&error_to_next == TRUE) { break }

}

}else{

while(TRUE){

message_to_next <<- TRUE

error_to_next <<- FALSE

try({withCallingHandlers(exp_clumped<-extract_instruments(outcomes = id,p1 = 1e-05,access_token = NULL),

message = function(c) if (stringr::str_detect(as.character(c),"Failed to")) message_to_next <<- FALSE)

error_to_next <<- TRUE})

if(message_to_next == TRUE&error_to_next == TRUE) { break }

}

}

table(!((((exp_clumped$beta.exposure)^2)/((exp_clumped$se.exposure)^2))<10))

exp_clumped <- exp_clumped[!((((exp_clumped$beta.exposure)^2)/((exp_clumped$se.exposure)^2))<10),]

dat<-list()

for (i in 1:length(id_exp$id)) {

print(id_exp$id[i])

while(TRUE){

message_to_next <<- TRUE

error_to_next <<- FALSE

try({withCallingHandlers(outcome<-extract_outcome_data(exp_clumped$SNP,

outcomes = id_exp$id[i],

proxies = F,

access_token = NULL),

message = function(c) if (stringr::str_detect(as.character(c),"Failed to")) message_to_next <<- FALSE)

error_to_next <<- TRUE})

if(message_to_next == TRUE&error_to_next == TRUE) { break }

}

if(is.null(outcome))next

dat[[i]]<-harmonise_data(exposure_dat = exp_clumped,outcome_dat = outcome)

}

dat<-do.call(rbind,dat)

dat<-dat[dat$mr_keep,]

write.csv(dat,file = "re_dat.csv",row.names = F)

re_res<-mr(dat)

write.csv(re_res,file = "re_res.csv",row.names = F)

#Heterogeneity analysis

heterogeneity<-mr_heterogeneity(dat)

write.csv(heterogeneity,file = "re_heterogeneity.csv",row.names = F)

# pleiotropy_test

pleiotropy_test<-mr_pleiotropy_test(dat)

write.csv(pleiotropy_test,file = "re_pleiotropy_test.csv",row.names = F)

# MRPRESSO

mp<-list()

mps<-unique(dat$id.exposure)

i=1

for (mpi in mps) {

data<-dat[dat$id.exposure==mpi,]

if(nrow(data)<4){next}

# MRPRESSO

mp[[i]]<-mr_presso(BetaOutcome = "beta.outcome",

BetaExposure = "beta.exposure",

SdOutcome = "se.outcome",

SdExposure = "se.exposure",

OUTLIERtest = TRUE,

DISTORTIONtest = TRUE,

data = data,

NbDistribution = 1000,

SignifThreshold = 0.05)

mp[[i]]$phe <- data$exposure[1]

i=i+1

}

result_mp_all<- data.frame()

for (i in 1:length(mp)) {

print(mp[[i]]$phe)

result_mp <- mp[[i]]$`Main MR results`

result_mp$RSSobs <- mp[[i]]$`MR-PRESSO results`$`Global Test`$RSSobs

result_mp$Pvalue <- mp[[i]]$`MR-PRESSO results`$`Global Test`$Pvalue

result_mp$phe <- mp[[i]]$phe

result_mp_all <- rbind(result_mp_all,result_mp)

}

table(is.na(result_mp_all$`Causal Estimate`))

write.csv(result_mp_all,file = "re_MRPRESSO.csv")

# draw

res_id<-read.csv("./res.csv")%>%get_sbeta_res()%>%dplyr::filter(method=="Inverse variance weighted",pval<0.05)%>%pull(id.exposure)

dat_total<-read.csv("./dat.csv")

for (idr in res_id) {

dat<-subset(dat_total,id.exposure==idr)

scatter_plot<-mr_scatter_plot(mr(dat),dat)[[1]]+

scale_color_lancet()+

scale_fill_lancet()+

theme(axis.title.y = element_text(size = 20))+

theme_bw(base_size = 16)+ theme(

plot.margin = margin(0.5,0.5,0.5,0.5, unit = "cm")

)

ggsave(plot=scatter_plot,filename = paste0(idr,"_scatter_plot_plot.pdf"),device = "pdf",width = 10,height = 10)

funnel_plot<-mr_funnel_plot(mr_singlesnp(dat,all_method=c("mr_egger_regression","mr_weighted_median","mr_ivw","mr_simple_mode","mr_weighted_mode")))[[1]]+

scale_color_lancet()+

scale_fill_lancet()+

theme(axis.title.y = element_text(size = 20))+

theme_bw(base_size = 16)+ theme(

plot.margin = margin(0.5,0.5,0.5,0.5, unit = "cm")

)

ggsave(plot=funnel_plot,filename = paste0(idr,"_funnel_plot_plot.pdf"),device = "pdf",width = 10,height = 10)

forest_plot<-mr_forest_plot(mr_singlesnp(dat))[[1]]+

scale_color_lancet()+

scale_fill_lancet()+

theme_bw()+

theme(legend.position = 'none',plot.margin = margin(0.5,0.5,0.5,0.5, unit = "cm"))

ggsave(plot=forest_plot,filename = paste0(idr,"_forest_plot_plot.pdf"),device = "pdf",width = 10,height = 10)

leaveoneout_plot<-mr_leaveoneout_plot(mr_leaveoneout(dat))[[1]]+

scale_color_lancet()+

scale_fill_lancet()+

theme_bw()+

theme(legend.position = 'none',plot.margin = margin(0.5,0.5,0.5,0.5, unit = "cm"),

axis.title.x = element_text(size = 12))

ggsave(plot=leaveoneout_plot,filename = paste0(idr,"_leaveoneout_plot_plot.pdf"),device = "pdf",width = 10,height = 10)

}

res_sign<-read.csv("./res.csv")%>%dplyr::filter(id.exposure%in%res_id)

res_sign$exposure<-str_split(str_split(res_sign$exposure,regex("\\(|\\)"),simplify = T)[,2]," id.",simplify = T)[,1]

res_sign$`OR (95% CI)` <- sprintf("%.3f (%.3f - %.3f)",res_sign$or, res_sign$or_lci95, res_sign$or_uci95)

res_sign$outcome[duplicated(res_sign$outcome)]<-""

dt<-res_sign[,c(3:6,9,12:14,15)]

dt$` ` <- paste(rep(" ", 30), collapse = " ")

dt$pval<-round(dt$pval,digits = 3)

dt$pval<-ifelse(dt$pval<0.001,"<0.001",dt$pval)

colnames(dt)[5]<-"italic(P)*-Value"

dt<-dt%>%dplyr::rename(Outcome=outcome,

`Trait`=exposure,

Method=method,

nSNP=nsnp,)

dt$Method<-ifelse(dt$Method=="Inverse variance weighted","IVW",dt$Method)

tm <- forest_theme(base_size = 8,colhead=list(fg_params = list(parse=TRUE)))

p=forest(dt[,c(2:5,10,9)], est = dt$or,

lower = dt$or_lci95,

upper = dt$or_uci95,

sizes = 0.6,

ci_column = 5,

ref_line = 1,

xlim = c(1,2),

ticks_at = c(4,6,8,10,12),

theme = tm)

ggsave("1.pdf",plot=p,device = "pdf",width = 20,height = 20)

######**2. Inflammatory proteins to disease**

library(dplyr)

library(MRPRESSO)

library(TwoSampleMR)

library(dplyr)

library(ggsci)

library(ggplot2)

library(ggpubr)

library(ggview)

library(plotly)

library(TSMRhelper)

library(forestploter)

library(grid)

library(stringr)

library(grDevices)

library(vroom)

library(gwasrapidd)

# outcome

#1.Online outcome

id<-"ebi-a-GCST90018916"

#2.Local outcome

id<-""

outcome_all<-fread("x.gz",data.table = F)

# Gets the directory of the exposed data set

getwd()

exposure_file <- dir("./data_p5e06/")

exp_clumped<-list()

for (i in 1:length(exposure_file)) {

exp_data<-read.csv(paste0("./data_p5e06/",exposure_file[i]))

out <- ieugwasr::ld_clump(dplyr::tibble(rsid=exp_data$SNP, pval=exp_data$pval.exposure,id = exp_data$id.exposure),

clump_kb = 10000,

clump_r2 = 0.001,

clump_p = 1,

plink_bin = plinkbinr::get_plink_exe(),

bfile = "D:/My R/Data cleaning/Local clump/1kg.v3/1kg.v3/EUR",

pop = "EUR")

keep <- paste(exp_data$SNP,exp_data$id.exposure) %in% paste(out$rsid,out$id)

exp_clumped[[i]] <- exp_data[keep,]

}

exp_clumped<-do.call(rbind,exp_clumped)

write.csv(exp_clumped,file = "91exp_clumped.csv",row.names=F)

# Get outcome

if(id==""){

snp<-unique(exp_clumped$SNP)

outcome<-outcome_all[outcome_all$rsids%in%snp,]

outcome_dat<-format_data(outcome,

type = "outcome",

beta_col = "beta",

se_col = "sebeta",

effect_allele_col = "alt",

other_allele_col = "ref",

snp_col = "rsids",

pval_col = "pval"

)

}else{

# This is the data to be modified by the online ending

while(TRUE){

message_to_next <<- TRUE

error_to_next <<- FALSE

try({withCallingHandlers(outcome_dat <- extract_outcome_data(snps = exp_clumped$SNP,

outcomes = id,

proxies = F,

access_token = NULL

),

message = function(c) if (stringr::str_detect(as.character(c),"Failed to")) message_to_next <<- FALSE)

error_to_next <<- TRUE})

if(message_to_next == TRUE&error_to_next == TRUE) { break }

}

}

dat <- harmonise_data(exposure_dat = exp_clumped,outcome_dat = outcome_dat)

dat<-dat[dat$mr_keep,]

write.csv(dat,file = "91_dat.csv",row.names = F)

# mr

res<-generate_odds_ratios(mr(dat))

write.csv(res,file = "91_res.csv",row.names = F)

# heterogeneity

heterogeneity<-mr_heterogeneity(dat)

write.csv(heterogeneity,file = "91_heterogeneity.csv",row.names = F)

# pleiotropy

pleiotropy_test<-mr_pleiotropy_test(dat)

write.csv(pleiotropy_test,file = "91_pleiotropy_test.csv",row.names = F)

# MRPRESSO

mp<-list()

mps<-unique(dat$id.exposure)

i=1

for (mpi in mps) {

data<-dat[dat$id.exposure==mpi,]

if(nrow(data)<4){next}

# MRPRESSO

mp[[i]]<-mr_presso(BetaOutcome = "beta.outcome",

BetaExposure = "beta.exposure",

SdOutcome = "se.outcome",

SdExposure = "se.exposure",

OUTLIERtest = TRUE,

DISTORTIONtest = TRUE,

data = data,

NbDistribution = 1000,

SignifThreshold = 0.05)

mp[[i]]$phe <- data$exposure[1]

i=i+1

}

result_mp_all<- data.frame()

for (i in 1:length(mp)) {

print(mp[[i]]$phe)

result_mp <- mp[[i]]$`Main MR results`

result_mp$RSSobs <- mp[[i]]$`MR-PRESSO results`$`Global Test`$RSSobs

result_mp$Pvalue <- mp[[i]]$`MR-PRESSO results`$`Global Test`$Pvalue

result_mp$phe <- mp[[i]]$phe

result_mp_all <- rbind(result_mp_all,result_mp)

}

table(is.na(result_mp_all$`Causal Estimate`))

write.csv(result_mp_all,file = "91_MRPRESSO.csv")

####### Reverse mr######

if(id==""){

exp<-outcome_all

exp$pval<-as.numeric(exp$pval)

exp<-subset(exp,pval<1e-05)

exp<-format_data(exp,

snp_col = "rsids",

effect_allele_col = "alt",

other_allele_col = "ref",

pval_col = "pval",

beta_col = "beta",

se_col = "sebeta")

while(TRUE){

message_to_next <<- TRUE

error_to_next <<- FALSE

try({withCallingHandlers(exp_clumped<-clump_data(exp),

message = function(c) if (stringr::str_detect(as.character(c),"Failed to")) message_to_next <<- FALSE)

error_to_next <<- TRUE})

if(message_to_next == TRUE&error_to_next == TRUE) { break }

}

}else{

while(TRUE){

message_to_next <<- TRUE

error_to_next <<- FALSE

try({withCallingHandlers(exp_clumped<-extract_instruments(outcomes = id,p1 = 1e-05,access_token = NULL),

message = function(c) if (stringr::str_detect(as.character(c),"Failed to")) message_to_next <<- FALSE)

error_to_next <<- TRUE})

if(message_to_next == TRUE&error_to_next == TRUE) { break }

}

}

table(!((((exp_clumped$beta.exposure)^2)/((exp_clumped$se.exposure)^2))<10))

exp_clumped <- exp_clumped[!((((exp_clumped$beta.exposure)^2)/((exp_clumped$se.exposure)^2))<10),]

write.csv(exp_clumped,"exp_clumped.csv")

# outcome

total_data<-dir("./91data/")

dat<-list()

for (i in 1:length(total_data)) {

print(total_data[i])

outcome_dat<-fread(paste0("./91data/",total_data[i]))

outcome_dat$p_value<-as.numeric(outcome_dat$p_value)

outcome_dat <- outcome_dat[!is.na(outcome_dat$rsid),]

outcome_dat<-subset(outcome_dat,rsid%in%unique(exp_clumped$SNP))

outcome_dat$id <- str_split(total_data[i],".tsv.gz",simplify = T)[1]

outcome_dat$phe<-get_studies(study_id = str_split(total_data[i],".tsv.gz",simplify = T)[1])@studies[["reported_trait"]]

outcome_dat<-format_data(outcome_dat,

type = "outcome",

snp_col = "rsid",

beta_col = "beta",

se_col = "standard_error",

eaf_col = "effect_allele_frequency",

effect_allele_col = "effect_allele",

other_allele_col = "other_allele",

pval_col = "p_value",

samplesize_col = "n",

chr_col = "chromosome",

pos_col = "base_pair_location",

id_col = "id",

phenotype_col = "phe")

dat[[i]]<-harmonise_data(exposure_dat = exp_clumped,outcome_dat = outcome_dat)

}

dat<-do.call(rbind,dat)

dat<-dat[dat$mr_keep,]

write.csv(dat,file = "91_re_dat.csv",row.names = F)

re_res<-mr(dat)

write.csv(re_res,file = "91_re_res.csv",row.names = F)

heterogeneity<-mr_heterogeneity(dat)

write.csv(heterogeneity,file = "91_re_heterogeneity.csv",row.names = F)

# pleiotropy_test

pleiotropy_test<-mr_pleiotropy_test(dat)

write.csv(pleiotropy_test,file = "91_re_pleiotropy_test.csv",row.names = F)

# draw

res_id<-read.csv("91_res.csv")%>%get_sbeta_res()%>%dplyr::filter(method=="Inverse variance weighted",pval<0.05)%>%pull(id.exposure)

dat_total<-read.csv("./91_dat.csv")

for (idr in res_id) {

dat<-subset(dat_total,id.exposure==idr)

scatter_plot<-mr_scatter_plot(mr(dat),dat)[[1]]+

scale_color_lancet()+

scale_fill_lancet()+

theme(axis.title.y = element_text(size = 20))+

theme_bw(base_size = 16)+ theme(

plot.margin = margin(0.5,0.5,0.5,0.5, unit = "cm")

)

ggsave(plot=scatter_plot,filename = paste0(idr,"_scatter_plot_plot.pdf"),device = "pdf",width = 10,height = 10)

funnel_plot<-mr_funnel_plot(mr_singlesnp(dat,all_method=c("mr_egger_regression","mr_weighted_median","mr_ivw","mr_simple_mode","mr_weighted_mode")))[[1]]+

scale_color_lancet()+

scale_fill_lancet()+

theme(axis.title.y = element_text(size = 20))+

theme_bw(base_size = 16)+ theme(

plot.margin = margin(0.5,0.5,0.5,0.5, unit = "cm")

)

ggsave(plot=funnel_plot,filename = paste0(idr,"_funnel_plot_plot.pdf"),device = "pdf",width = 10,height = 10)

forest_plot<-mr_forest_plot(mr_singlesnp(dat))[[1]]+

scale_color_lancet()+

scale_fill_lancet()+

theme_bw()+

theme(legend.position = 'none',plot.margin = margin(0.5,0.5,0.5,0.5, unit = "cm"))

ggsave(plot=forest_plot,filename = paste0(idr,"_forest_plot_plot.pdf"),device = "pdf",width = 10,height = 10)

leaveoneout_plot<-mr_leaveoneout_plot(mr_leaveoneout(dat))[[1]]+

scale_color_lancet()+

scale_fill_lancet()+

theme_bw()+

theme(legend.position = 'none',plot.margin = margin(0.5,0.5,0.5,0.5, unit = "cm"),

axis.title.x = element_text(size = 12))

ggsave(plot=leaveoneout_plot,filename = paste0(idr,"_leaveoneout_plot_plot.pdf"),device = "pdf",width = 10,height = 10)

}

# Forest map

res_sign<-read.csv("91_res.csv")%>%dplyr::filter(id.exposure%in%res_id)

res_sign$`OR (95% CI)` <- sprintf("%.3f (%.3f - %.3f)",res_sign$or, res_sign$or_lci95, res_sign$or_uci95)

res_sign$outcome[duplicated(res_sign$outcome)]<-""

dt<-res_sign[,c(3:6,9,12:14,15)]

dt$` ` <- paste(rep(" ", 30), collapse = " ")

dt$pval<-round(dt$pval,digits = 3)

dt$pval<-ifelse(dt$pval<0.001,"<0.001",dt$pval)

colnames(dt)[5]<-"italic(P)*-Value"

dt<-dt%>%dplyr::rename(Outcome=outcome,

`Trait`=exposure,

Method=method,

nSNP=nsnp,)

dt$Method<-ifelse(dt$Method=="Inverse variance weighted","IVW",dt$Method)

tm <- forest_theme(base_size = 8,colhead=list(fg_params = list(parse=TRUE)))

p=forest(dt[,c(2:5,10,9)],

est = dt$or,

lower = dt$or_lci95,

upper = dt$or_uci95,

sizes = 0.3,

ci_column = 5,

ref_line = 1,

xlim = c(0.5,1.5),

ticks_at = c(0.8,0.9,1.0,1.1,1.2),

theme = tm)

ggsave("2.pdf",plot=p,device = "pdf",width = 20,height = 20)

######**3. Gut microbiome to inflammatory proteins**

library(TSMRhelper)

library(dplyr)

library(gwasrapidd)

library(vroom)

library(data.table)

gut_res<-read.csv("./res.csv")%>%get_sbeta_res()%>%dplyr::filter(method=="Inverse variance weighted",pval<0.05)

res91<-read.csv("./91_res.csv")%>%get_sbeta_res()%>%dplyr::filter(method=="Inverse variance weighted",pval<0.05)

gut_exp<-read.csv("exp_clumped.csv")%>%dplyr::filter(id.exposure%in%gut_res$id.exposure)

ids<-paste0(res91$id.exposure,".tsv.gz")

dat<-list()

for (i in 1:length(ids)) {

print(ids[i])

outcome_dat<-fread(paste0("./91data/",ids[i]),data.table=F)

outcome_dat$p_value<-as.numeric(outcome_dat$p_value)

outcome_dat <- outcome_dat[!is.na(outcome_dat$rsid),]

outcome_dat<-subset(outcome_dat,rsid%in%unique(gut_exp$SNP))

outcome_dat$id <- str_split(ids[i],".tsv.gz",simplify = T)[1]

outcome_dat$phe<-get_studies(study_id = str_split(ids[i],".tsv.gz",simplify = T)[1])@studies[["reported_trait"]]

outcome_dat<-format_data(outcome_dat,

type = "outcome",

snp_col = "rsid",

beta_col = "beta",

se_col = "standard_error",

eaf_col = "effect_allele_frequency",

effect_allele_col = "effect_allele",

other_allele_col = "other_allele",

pval_col = "p_value",

samplesize_col = "n",

chr_col = "chromosome",

pos_col = "base_pair_location",

id_col = "id",

phenotype_col = "phe")

dat[[i]]<-harmonise_data(exposure_dat = gut_exp,outcome_dat = outcome_dat)

}

dat<-do.call(rbind,dat)

dat<-dat[dat$mr_keep,]

write.csv(dat,file = "gutto91_dat.csv",row.names = F)

res<-mr(dat)

write.csv(res,file = "gutto91_res.csv",row.names = F)

res<-read.csv("gutto91_res.csv")

#### Could be the result of mediation

id_final<-res%>%dplyr::filter(method=="Inverse variance weighted",pval<0.05)

for (a in 1:nrow(id_final)) {

print(paste0("Exposure：",id_final[a,]$id.exposure,"mediation：",id_final[a,]$id.outcome))

}

######4. MR Analysis of Fenland protein for disease

setwd("F:/result")

library(dplyr)

library(TwoSampleMR)

library(MRPRESSO)

library(stringr)

library(parallel)

library(pbapply)

# Gets a list of all txt files

txt_files <- list.files(pattern = "\\.TXT$", full.names = TRUE)

combined_data <- data.frame()

# Loop through each txt file

for (file in txt_files) {

while (TRUE) {

data <- read.table(file, header = TRUE)

colnames(data) <- c("SNP", "ALT", "REF", "EAF", "BETA", "SE", "P", "N", "CHR", "POS")

file_name <- basename(file)

data <- mutate(data, Protein_file = file_name)

data <- filter(data, P < 5e-5)

data <- mutate(data, SNP = as.character(SNP), CHR = as.character(CHR), ALT = as.character(ALT), REF = as.character(REF))

data$id <- data$Protein_file

if (nrow(data) == 0) {

#If the result is empty, the next TXT file is read again

break

}

exp <- format_data(data,

phenotype_col = "Protein_file",

chr_col = "CHR",

pos_col = "POS",

snp_col = "SNP",

effect_allele_col = "ALT",

other_allele_col = "REF",

beta_col = "BETA",

se_col = "SE",

pval_col = "P",

eaf_col = "EAF",

samplesize_col = "N"

)

out <- try(ieugwasr::ld_clump(dplyr::tibble(rsid = exp$SNP, pval = exp$pval.exposure, id = exp$id.exposure),

clump_kb = 10000,

clump_r2 = 0.001,

clump_p = 1,

plink_bin = plinkbinr::get_plink_exe(),

bfile = "D:/My R/Data cleaning/Local clump/1kg.v3/1kg.v3/EUR",

pop = "EUR"), silent = TRUE)

if (inherits(out, "try-error") || nrow(out) == 0) {

message("No significant --clump results or an error occurred. Re-reading next file.")

break

}

keep <- paste(exp$SNP, exp$id.exposure) %in% paste(out$rsid, out$id)

exp <- exp[keep,]

combined_data <- bind_rows(combined_data, exp)

break

}

}

# Save the results as a large CSV file

write.csv(combined_data, "clumped_data_5e-5.csv", row.names = FALSE)

# Output completion message

cat("Merge and save successfully！\n")

fenlhe<-read.csv("fenland merges require files.csv")

combined_data1<-merge(combined_data,fenlhe,by.x ="exposure" ,by.y = "newname",all.x = TRUE)

write.csv(combined_data1, "clumped_data_5e-5.csv", row.names = FALSE)

exposure_file<-unique(combined_data1$exposure)

exp_clumped<-read.csv("clumped_data_5e-5.csv")

# Exclude F values (all F>=10)

table(!((((exp_clumped$beta.exposure)^2)/((exp_clumped$se.exposure)^2))<10))

# Import local outcome

library(vroom)

library(dplyr)

exp_clumped<-vroom("F:/result/clumped_data_5e-5.csv")

exp_clumped<-exp_clumped[,-1]

exp_clumped<-exp_clumped %>% rename(exposure=Target)

setwd("F:/Fenalnd MR")

outcome<-vroom("F:/X.csv")

outcome<- outcome[complete.cases(outcome[, 2]), ]

outcome<- TwoSampleMR::format_data(

outcome,

type = "outcome",

snp_col = "SNP",

beta_col = "BETA",

se_col = "SE",

eaf_col = "EAF",

effect_allele_col = "ALT",

other_allele_col = "REF",

pval_col = "P",

samplesize_col = "N"

)

dat<-harmonise_data(exp_clumped,outcome)

dat<-dat[dat$mr_keep,]

dat<-split(dat,dat$id.exposure) #Group by gene

# Start parallel computing

cl <- makeCluster(detectCores(logical = FALSE)-1)

system.time(

res<- parLapply(cl,dat,mr)

)

#Close parallel

stopCluster(cl)

# Combined result

res<-do.call(rbind,res)

rownames(res)<-NULL

table(res$method)

# Save the result

write.csv(res,file = "res.csv",row.names = F)

res <- read.csv("res.csv")

res$pval_adj <- p.adjust(res$pval, method = 'fdr')

res<-generate_odds_ratios(res)

write.csv(res,"res_p_adj.csv")

# Screened for IVW or Wald ratio exposures with P values less than 0.05

result <- read.csv("res.csv")

method <- result[result$method=="Inverse variance weighted"|result$method=="Wald ratio",]

id_final <- method[method$pval<0.05,]$id.exposure

if(length(id_final)==0){print("There were no significant results")}

# In the data after screening, multiple effects, heterogeneity and other operations were performed

res_total <- list()

i<-1

for (gene in id_final) {

print(gene)

data<-dat[[gene]]

# MR

res <- mr(data)

# heterogeneity

if(nrow(data)<2){res[,c("Q","Q_df","Q_pval")]<-NA}else{

res<- merge(res,mr_heterogeneity(data)[,c("method","Q","Q_df","Q_pval")],by = "method")}

# pleiotropy

res[res$method=="MR Egger",c("Egger_intercept","Pleiotropy_pval")]<-ifelse(nrow(data)<3,NA,mr_pleiotropy_test(data)%>%

dplyr::rename(Pleiotropy_pval=pval)%>%

dplyr::select(egger_intercept,Pleiotropy_pval))

res_total[[i]] <- res

i<-i+1

}

result_significance <- do.call(rbind,res_total)

write.csv(result_significance,file = "result_significance.csv")

# Running MRPRESSO takes a long time. Be careful!

######MRPRESSO######

mp<-list()

i=1

for (gene in id_final[1:length(id_final)]) {

print(gene)

data<-dat[[gene]]

if(nrow(data)<4)next

# MRPRESSO

mp[[i]]<-mr_presso(BetaOutcome = "beta.outcome",

BetaExposure = "beta.exposure",

SdOutcome = "se.outcome",

SdExposure = "se.exposure",

OUTLIERtest = TRUE,

DISTORTIONtest = TRUE,

data = data,

NbDistribution = 1000,

SignifThreshold = 0.05)

mp[[i]]$phe <- gene

i=i+1

}

result_mp <- data.frame()

result_mp_all <- data.frame()

for (i in 1:length(mp)) {

print(mp[[i]]$phe)

result_mp <- mp[[i]]$`Main MR results`

result_mp$RSSobs <- mp[[i]]$`MR-PRESSO results`$`Global Test`$RSSobs

result_mp$Pvalue <- mp[[i]]$`MR-PRESSO results`$`Global Test`$Pvalue

result_mp$bac <- mp[[i]]$phe

result_mp_all <- rbind(result_mp_all,result_mp)

}

table(is.na(result_mp_all$`Causal Estimate`))

write.csv(result_mp_all,file = "MRPRESSO.csv")

#####**5. Example code for hyprcoloc analysis**

library(hyprcoloc)

library(dplyr)

betas <- hyprcoloc::test.betas

head(betas)

ses <- hyprcoloc::test.ses

head(ses)

trait.cor <- hyprcoloc::test.corr

ld.matrix <- hyprcoloc::test.ld

# Trait names and SNP IDs

traits <- paste0("T", 1:10)

rsid <- rownames(betas)

#hyprcoloc analysis

hyprcoloc(betas, ses, trait.names=traits, snp.id=rsid)

#######Label traits as binary or continuous variables, with continuous variables encoded as 0 and categorical variables encoded as 1

binary.traits = c(1,1,1,rep(0,dim(betas)[2]-3))

res <- hyprcoloc(betas, ses, trait.names=traits, snp.id=rsid, binary.outcomes = binary.traits)

res

#####**6. Sample code for Colocalization analysis**

library(dplyr)

library(plyr)

library(data.table)

####eaf<0.5:eaf=maf eaf>0.5:maf=1-eaf

D1<-list(type="quant",

beta=data$slope,

varbeta=data$slope_se^2,

N=838,

MAF=data$maf,

snp=data$rs_id_dbSNP151_GRCh38p7)

D2<-list(type="cc",

beta=data$beta,

varbeta=data$se^2,

s=0.3,

N=838,

MAF=data$minor_AF,

snp=data$rs_id_dbSNP151_GRCh38p7)

#### COLOC.ABF #####

library(coloc)

coloc<-coloc.abf( D1, D2, p1 = 1e-04,p2 = 1e-04,p12 = 1e-05)

result=coloc$results
